# Analysis of Phenotypic and Molecular Variability of Memory-like NK Cells for Cancer Adoptive Cell Therapy Screening

**DOI:** 10.3390/cancers17142288

**Published:** 2025-07-09

**Authors:** Rithvik V. Turaga, Seth R. T. Zima, Ella P. Peterson, Amy K. Erbe, Matthew H. Forsberg, Christian M. Capitini, Pippa F. Cosper, Paul M. Sondel, Jose M. Ayuso

**Affiliations:** 1 Department of Dermatology, School of Medicine and Public Health, University of Wisconsin, 1 S Park Street, Madison, WI 53715, USA; rturaga@wisc.edu (R.V.T.); srzima@wisc.edu (S.R.T.Z.); eppeterson@wisc.edu (E.P.P.); 2 Department of Biomedical Engineering, College of Engineering, University of Wisconsin, 1550 Engineering Dr, Madison, WI 53706, USA; 3 UW Carbone Cancer Center, 600 Highland Avenue, Madison, WI 53705, USA; aerbe@wisc.edu (A.K.E.); ccapitini@pediatrics.wisc.edu (C.M.C.); cosper@humonc.wisc.edu (P.F.C.); pmsondel@humonc.wisc.edu (P.M.S.); 4 Department of Human Oncology, School of Medicine and Public Health, University of Wisconsin, Madison, WI 53705, USA; 5 Department of Pediatrics, School of Medicine and Public Health, University of Wisconsin, Madison, WI 53705, USA; mhforsberg@wisc.edu

**Keywords:** natural killer cells, memory-like natural killer cells, head and neck squamous cell carcinoma, immunotherapy, adoptive cell therapy, phenotypic and molecular variability, prediction

## Abstract

Background: Adoptive cell therapies are emerging as a promising therapeutic option against hematological and solid malignancies. Memory-like natural killer (mlNK) cells are a specific subtype of NK cells generated after cytokine preactivation that have shown enhanced in vivo persistence after infusion into patients, an issue that has hindered traditional NK cell immunotherapy. However, the quality and variability of mlNK cell products remains poorly defined. Methods: In this study, we evaluated heterogeneity across critical functional and molecular aspects of mlNK cells generated from independent donors, including mlNK cytotoxicity, cluster formation, motility, mitochondria morphology, and gene expression. Results: We observed a correlation between changes in gene expression associated with glycolysis and key NK cell functions such as cytotoxicity and motility. For further characterization, we blocked glycolysis and oxidative phosphorylation (OXPHOS) and observed an impaired mlNK functional response, suggesting the importance of metabolism. Conclusions: Our findings provide insights into discriminating between mlNK cell products and how the predictive markers can identify optimal mlNK cell products for adoptive cell therapy of cancer.

## Simple Summary

Adoptive cell therapy has emerged as an alternative cancer immunotherapeutic option. Memory-like natural killer (NK) cells are a type of immune cell that have shown promise due to their superior ability to persist in vivo after cytokine preactivation and exhibit a robust response upon antigen re-exposure. However, memory-like NK cells can vary in quality based on their functional and molecular characteristics. In this study, we studied functional heterogeneity across memory-like NK cell products generated from multiple donors. We observed that there were significant differences across several parameters for these NK cell products, most notably in their cytotoxic (killing) capacity against head and neck cancer cells. Memory-like NK cells that exhibited poor cytotoxicity also had the weakest motility and disrupted gene expression related to metabolism. Overall, our findings underscore the importance of characterizing memory-like NK cells to identify the most effective donor-derived products for adoptive cell therapy of cancer.

## 1. Introduction

Cancer immunotherapy has revolutionized the landscape of cancer treatment and management, cementing itself as a first-line treatment for many malignancies. Immunotherapies aim to boost or supplement a patient’s immune response to eliminate cancer cells [1]. Cancer immunotherapies can be further divided into several sub-categories such as oncolytic viral therapies like reovirus and adenovirus, cancer vaccines that utilize tumor-specific antigens to initiate an immune response, cytokine therapies like IL-2 and IFN-α that were initially used against melanoma, immune checkpoint inhibitors used to stimulate anti-tumor immune responses by disrupting inhibitory signals, and adoptive cell therapies (ACTs) which use ex vivo expanded and engineered immune cells [2,3,4,5,6,7].

Recently, ACT has emerged as an exciting therapeutic alternative against hematological malignancies and is also being explored against solid malignancies. In ACT, cells can be activated ex vivo against tumors without disruption from inhibitory signals released in the tumor microenvironment [7]. Many ACTs rely on the use of autologous T cells (i.e., cells derived from the cancer patient) rather than allogeneic (i.e., cells derived from a healthy donor and then infused into a cancer patient). ACT with allogeneic T cells is challenging due to donor–recipient mismatch of human leukocyte antigen (HLA) potentially leading to the onset of life-threatening graft-versus-host-disease. [8]. However, there are numerous scenarios where ACT with autologous cells is not possible (e.g., patients with lymphopenia). Therefore, novel approaches to optimizing allogeneic ACTs may offer a promising alternative, or complement, to autologous ACT.

Natural killer (NK) cells are a promising ACT because they do not cause graft-versus-host-disease, despite HLA mismatch, allowing for their use as allogeneic therapies. NK cells have also been shown to be an effective strategy against several tumors [9]. In the case of head and neck cancer, recent clinical trials of patients treated with immune checkpoint inhibitor therapy have not shown promising results, implying a significant unmet clinical need to enhance the anti-tumor immune response [10,11]. NK cell ACT has emerged as a potential alternative or complementary approach [12]. NK cells are cytolytic immune cells that have the capacity to kill tumor cells via several mechanisms including the expression of pro-apoptosis ligands (e.g., FasL) and the secretion of cytotoxic proteins (e.g., granzymes) [13]. The therapeutic potential of NK cells in treating head and neck cancer has been supported by several studies showing a positive correlation between patient prognosis and NK cell infiltration in head and neck cancer squamous cell carcinoma (HNSCC) [14,15,16].

A limitation of ACT with NK cells is limited in vivo persistence, which severely decreases their impact on inducing a durable clinical anti-tumor response. Recent studies have indicated that cytokine-preactivated NK cells can be further differentiated into a memory-like phenotype, allowing for a quicker and more robust response to tumors and significantly elongated persistence after ACT to patients [17,18]. Therefore, these memory-like NK cells (mlNK) offer a novel avenue for ACT. Traditionally, killer immunoglobulin receptor (KIR)/KIR-ligand genotyping has provided a way to predict NK cell anti-tumor efficacy. However, KIR/KIR-ligand screening struggles to capture all the complexity of NK cell anti-tumor responses, and the novelty of mlNK emphasizes this limitation.

In addition, we have limited understanding regarding the functional variability of mlNK cell products. Some previous studies have shown that in vivo, mlNK cells undergo dynamic changes such as increased frequency of inhibitory receptors that make them distinct from ex vivo-activated NK cells. However, this study was focused on molecular differences [19]. In another study, optimal culture conditions for mlNK cell expansion and cytotoxicity were evaluated for future clinical trials. However, a large-scale functional characterization was not performed in this study [20]. In both of these studies, single-cell heterogeneity analyses to evaluate intra donor variability were excluded. In this study, we evaluate inter- and intra-patient heterogeneity, highlighting the need for single-cell and high-dimensional characterization across multiple orthogonal readouts to determine ideal donor candidates and NK cell populations for mlNK ACT.

Primary human NK cells were isolated and preactivated into mlNK cells to elucidate the variability in their behavior. We first evaluated their cytotoxic ability against the HNSCC cell line FaDu-HTB-43 (referred to as Fadu). This was followed by an analysis of KIR-KIR ligand genotyping, cellular expansion or contraction over the course of memory-like NK cell generation, viability, activation markers such as cluster formation, motility analyses, mitochondrial structure, and genetic expression data to elucidate common trends that could predict mlNK cell cytotoxicity.

## 2. Materials and Methods

### 2.1. NK Cell Isolation

The NK cells used for this experiment were isolated from human donor peripheral blood (IRB-exempt) purchased from Leukocyte Reduction System cones (Versiti Blood Bank, Milwaukee, WI, USA). All patients in the donor pool were over the age of 50 years and were tested for common infectious diseases such as—Human Immunodeficiency Virus (HIV), Human T-cell lymphotropic virus (HTLV), Syphilis, Hepatitis B and C, Cytomegalovirus (CMV), West Nile, and Trypanosoma Cruzi (Table S2). The blood was transferred into a 50 mL tube (Ref: 91051; TPP) and then diluted 1:1 with 1× phosphate-buffered saline (PBS, Ref: 14190-144; Gibco, Waltham, MA, USA). The diluted blood was then slowly added to a separate tube containing 15mL Lymphoprep (Ref: 07801; Stem Cell, Vancouver, BC, Canada). The tube was centrifuged for 30 min at 800× *g* with no brake (Ref: ST16R; Thermo Scientific, Waltham, MA, USA). Once the centrifugation was complete, the peripheral blood mononuclear cell (PBMC) layer was transferred to a separate tube. A total of 5 mL of Red Blood Cell (RBC) lysis buffer (Ref: 00-4333-57; Invitrogen, Waltham, MA, USA) was added and incubated with the PBMCs and mixed continuously for 5 min. The solution was made up to 50 mL with 1× PBS and centrifuged for 5 min at 400× *g*. This process was repeated until the pellet was no longer red. PBMCs were then collected and counted for further experiments. NK cells were isolated from the PBMCs using negative selection by magnetic cell separation (Ref: 130-092-657; Miltenyi Biotec, North Rhine-Westphalia, Germany).

### 2.2. Memory-like NK Cell Generation and Culture

NK cells isolated from blood were cultured in memory media for 1 day, after which the media were changed to basal media. Basal media were prepared by adding 5 mL Human AB Serum (Ref: 2931949; MP Biomedicals, Solon, OH, USA) and 0.25 µL of 200 µg/mL IL-15 (Ref: 200-15-50UG; Peprotech, Rocky Hill, NJ, USA) to 45 mL of X-VIVO 15 (Ref: BEBP04-744Q; Lonza, Basel, Switzerland). The memory media were made of 10 mL basal media, 0.5 µL 200 µg/mL IL-12 (Ref: 200-12H-10UG; Peprotech), 2.5 µL 200 µg/mL IL-15, and 2.5 µL 200 µg/mL IL-18 (Ref: 912-IL/CF; R&D Systems, Minneapolis, MN, USA). Naive NK cells were cultured in memory media for 1 day at a density of 5 × 10^6^ cells/ mL in flasks. The media were then changed to basal media and cells were incubated for 6 days at 37 °C and 5% CO_2_.

### 2.3. Flow Cytometry

Naïve and memory-like NK cells were collected and stained at 4 °C for 15–20 min with anti-human antibodies for CD16-PE-Cy5, CD56-BV510, NKG2A-PE-Cy7, and NKp46-PE-Cy7 (Biolegend, San Diego, CA, USA). Samples were run on an Attune Flow Cytometer (ThermoFisher Scientific), converted to FCS files, and then analyzed using FlowJo software (Version 10.9 | FlowJo, LLC).

### 2.4. Fadu Tumor Cell Culture

FaDu-HTB-43 cells were gifted by Dr. Pippa Cosper (University of Wisconsin-Madison). Tumor cells were seeded in a cell culture dish (1100 × 20 mm, Ref: 30702115; Eppendorf, Hamburg, Germany)) and cultured with Dulbecco’s modified eagle medium (DMEM; 11965-092; Gibco, Waltham, MA, USA) supplemented with 10% fetal bovine serum (FBS, Ref: 35-010-CV; Corning, Corning, NY, USA) and 5% penicillin–streptomycin (10,000 U/mL, Ref: 15140122; Gibco). Fadu cells were grown in a humidified cell incubator (5% CO_2_ and 37 °C). Upon reaching 70–80% confluency, cells were lifted, counted, and resuspended at the required density for experimental purposes.

### 2.5. Cytotoxicity

Fadu tumor cells were lifted and stained using 10 mM CellTracker Green CMFDA (Ref: C7025; Invitrogen), as previously described. Fadu cells were seeded at the desired density in a 96-well plate (Ref: 165300; Thermo Scientific) and allowed to culture at 37 °C and 5% CO_2_ to allow for proper adhesion. The media were then removed from wells containing Fadu cells and replaced with media containing mlNK cells at the necessary densities (densities were determined to allow for a 1:1 and 1:8 effector/target ratio). To observe the baseline cell death of Fadu cells, a control group was set up without any mlNK cells. The cells were cultured for 3 days at 37 °C and 5% CO_2_ and imaged using a Leica Microscope (Thunder Imager, Leica DMi8, Leica, Wetzlar, Germany). Cytotoxicity was analyzed by comparing the % area occupied by viable Fadu cells, using the Fiji image processing software (Version 1.8.0).

### 2.6. Genotyping Analysis

KIR2DL1, 2DL2, 2DL3, and 3DL1 were genotyped by a SYBR green real time PCR reaction, and KIR-ligands (HLA-C1, C2, and Bw4) were genotyped using the KIR HLA Ligand SSP typing kit (CareDx, Brisbane, CA, USA), as previously described [21].

### 2.7. mlNK Cell Viability

NK cell viability was assessed after memory induction on day 7 with the help of two-color fluorescent dyes, 10 mM cell tracker green (CMFDA; Ref: C7025; ThermoFisher Scientific) and 2 mg/mL propidium iodide (PI; Ref: P4170-25MG; Millipore Sigma, Burlington, MA, USA), prepared in DMSO (Dimethyl Sulfoxide; Ref: 67-68-5; Santa Cruz Biotechnology, Dallas, TX, USA) and distilled water, respectively. Working solutions were prepared by diluting stocks in 1× PBS at 1:1000 for both stains. mlNK cells were stained in cell tracker and PI for 15 min in a humidified cell incubator (5% CO_2_ and 37 °C). Next, the cells were washed twice with 10 mL of 1× PBS to remove excess staining. Cells were re-plated in a 96-well plate and imaged using a Leica Microscope (Thunder Imager, Leica DMi8). The viable and dead cell number was quantified using the Fiji image processing software.

### 2.8. Clustering

Throughout the memory protocol, images were obtained from NK cells over a 7-day period. The images were taken on day 0, day 1, day 3, and day 7 using a brightfield microscope (Thunder Imager, Leica DMi8). The cluster size and number were analyzed using the Fiji image processing software.

### 2.9. Motility Analysis

Motility analysis was conducted on mlNK cells after 7 days of memory protocol. mlNK cells were stained with cell tracker green as previously described. The stained cells were plated in a 96-well plate (Ref: 165300; Thermo Scientific). The cells were imaged for 30 min every 15 s to obtain a timelapse video depicting their motility. These videos were captured using a Leica Microscope (Thunder Imager, Leica DMi8). The videos were then processed using the Trackmate plugin on the Fiji image processing software.

### 2.10. Single-Cell Computational Analysis

UMAP plots were generated in Python v3.12.3 using the package ‘umap.’ The optimal number of clusters for k-means was identified using within cluster sum of squares (WCSS).

### 2.11. Mitochondrial Staining and Analysis

After induction into memory-like NK cells, the cells were analyzed for their mitochondrial mass and structure. To visualize mitochondria, the cells were stained using MitoTracker Red CMXRos (Ref: 9082; Cell Signaling Technology, Danvers, MA, USA). A 1mM stock solution was prepared by reconstituting in 94.1 µL of DMSO. The cells were incubated in a 50 nM working solution (prepared by dilution in 1× PBS) for 30 min at 37 °C and 5% CO_2_. The cells were then washed twice with 1× PBS and then plated on a glass bottom 96-well plate (Ref: 165306; ThermoScientific) for image acquisition using a Leica Microscope (Thunder Imager, Leica DMi8). Images were analyzed using the MorphoLibJ plugin in Fiji.

### 2.12. Gene Expression Analysis

RT-qPCR was conducted to the evaluate gene expression profiles of mlNK cells for all donors. After the memory induction protocol, the cells were collected, and their RNA was extracted using Qiagen RNeasy Plus Micro Kit (Ref: 74034; Qiagen, Hilden, Germany). Complementary DNA (cDNA) was generated using a RT^2^ PreAMP cDNA Synthesis Kit (Ref: 330451; Qiagen) and mixed with RT^2^ SYBR Green Fluor qPCR Mastermix (Ref: 330513; Qiagen) and RNase-free water, as recommended. The resulting mixture was aliquoted into a Glucose Metabolism RT^2^ Profiler PCR Array (Ref: 330231; Qiagen) for PCR. The relative fold change in gene expression was determined using the 2^ΔΔCt^ method and normalized to the internal controls. Gene expression data are publicly available in the Gene Expression Omnibus by NCBI (Accession number: GSE296965) (https://www.ncbi.nlm.nih.gov/geo/query/acc.cgi?acc=GSE296965 (accessed on 13 May 2025)).

### 2.13. Two-Dimensional and Oligomycin Exposure

NK cells were isolated from human donor peripheral blood, as described earlier. NK cells were split into 4 conditions during the memory protocol—exposed to media with 50 mM 2-DG (Ref: HY-13966-1G; Fisher Scientific), exposed to 2-DG vehicle control media, exposed to 100 nM oligomycin (Ref: 495455-10MG; Sigma Aldrich), and exposed to oligomycin vehicle control media. After 7 days in each respective media, cells were analyzed for their cytotoxicity, viability, clustering, motility, and mitochondria morphology with the previously described methods.

### 2.14. Image Acquisition and Analysis

All images were obtained using a fluorescence microscope, the Leica Thunder Imager (Model DMi8 automated). A stage-top incubator at 37 °C and 5% CO_2_ was used when imaging live cells. The images taken using the Thunder Imager were processed and analyzed using the Fiji software.

### 2.15. Equipment

All microscopy images were acquired using a Thunder Imager Microscope DMi8 (Leica, Wetzlar, Germany). RT-qPCR was performed on Light Cycler 480 (Roche, Basel, Switzerland). Flow analysis was performed on Attune Flow Cytometer (ThermoFisher Scientific, Waltman, MA, USA).

### 2.16. Statistical Analysis

Statistical analysis was performed on GraphPad Prism 9. A one-way ANOVA analysis was used for all parametric comparisons. A *p* value of <0.05 was set to determine statistical significance. All experiments conducted included at least 3 biological replicates.

## 3. Results

### 3.1. Differences in Cytotoxic Potential Across mlNK Products Showcases the Need for Further Characterization

**Figure 1:**
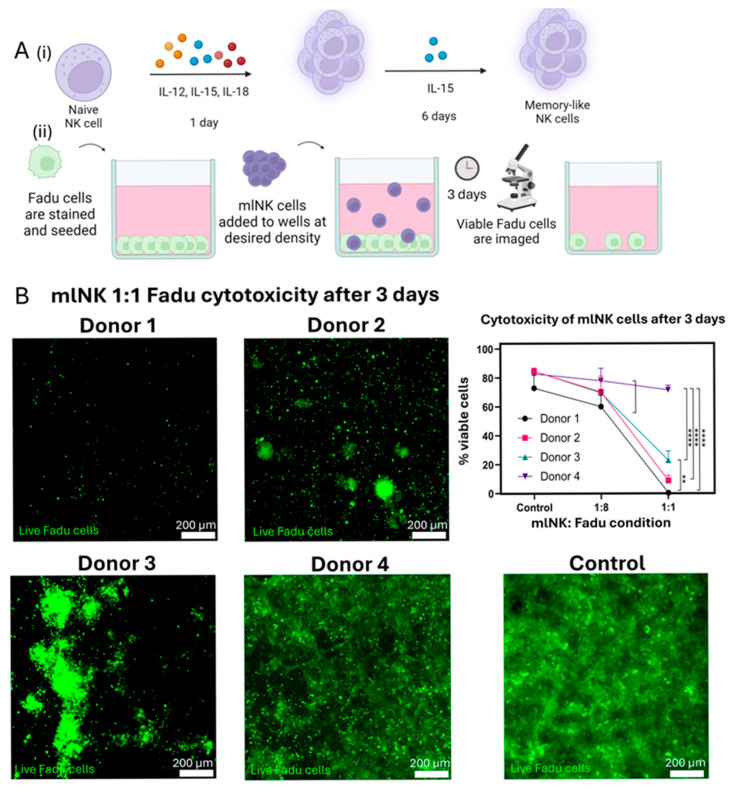
(**A**) Schematic overview of the protocol used to (**i**) generate mlNK cells and (**ii**) experimental setup to measure the cytotoxicity of mlNK cells. (**B**) Fluorescent images of viable tumor cells after exposure to mlNK cells at a 1:1 ratio over 72 h for all four donors and a control sample (no NK cells). Graphical quantification showing differences in cytotoxicity for all conditions and donors. Graph shows average ± standard deviation. ** and **** represent *p*-value < 0.01, and <0.0001, respectively.

Naïve NK (nNK) cells were isolated from PBMCs from four healthy donors and preactivated with an IL-12, IL-15, and IL-18 cytokine cocktail for 24 h, after which the media were changed to basal media (50 mL X-VIVO 15 supplemented with 0.25 µL of 200 µg/mL IL-15). The cells were then expanded with IL-15 over the course of 6 days to enrich for a memory-like NK phenotype (Figure 1A(i)) [22]. Flow cytometric analysis showed that mlNK cells have a higher expression of CD56, NKG2A, and NKp46 and lower expression of CD16 compared to nNK cells (Figure S1). These data are consistent with previous studies performed on mlNK cells. In addition, intra- and inter-patient variability in mlNK cell population for NKG2A and NKp46 was observed. Cytotoxicity is one of the most important functional attributes of NK cells in the context of ACT. mlNK cells were seeded on top of a Fadu cell monolayer at different NK; tumor cell ratios (1:1 and 1:8) for 72 h and tumor viability was measured (Figure 1A(ii) and Figure S2). The data indicated a decrease in tumor cell area as the number of mlNK cells added to the wells increased. The 1:1 condition saw the largest reduction in total viable tumor cells in all donors. We observed a trend in donor toxicity in decreasing order, as follows: Donor #1 > #2 > #3 > #4. Donor #4 was the largest outlier, with a marginal decrease in tumor cell viability compared to the control (no mlNK cells) with a statistically significant reduction in cytotoxicity compared to all other donors (Figure 1B). To better understand variables potentially driving the differences observed in cytotoxicity, first we determined if KIR genotyping analysis could verify the results obtained from the cytotoxicity experiments.

### 3.2. KIR/KIR-Ligand Genotyping Is Not Sufficient to Accurately Predict Phenotypic Heterogeneity

NK cell effector functions are, at least partially, regulated by the interaction between major histocompatibility complex (MHC) class I molecules and their inhibitory receptors such as KIRs. This interaction can result in the activation or inhibition of NK cell activity. During the development and maturation of NK cells, the interaction between KIRs and their ligands is crucial for cells to become functionally competent. This education process is known as NK cell licensing and ensures that NK cells effectively distinguish between healthy cells and cancerous or infected cells [23,24]. We considered whether individual genotypic differences in KIRs (KIR2DL1, KIR2DL2, KIR2DL3, and KIR3DL1) and their ligands (HLA-C1, HLA-C2, and HLA-Bw4) might impact the killing capabilities of mlNK cells. KIR2DL1 is a receptor for HLA-C2, KIRs 2DL2/2DL3 are receptors for HLA-C1, and KIR3DL1 is a receptor for HLA-Bw4 [25,26,27]. Each of these receptor/ligand interactions influences NK cell licensing (endogenously within the donor) and the potential for inhibition of NK cell killing capabilities against Fadu cells (NK cell to tumor cell interactions) [28,29,30]. Fadu cells express MHCI on their surface (Figure S3) and are positive for HLA-C1 and HLA-Bw4 but lack HLA-C2 (Supplemental Table S1). Donors 1 and 2 were identical for their KIR genes and KIR-ligands, and had NK cells that expressed KIR2DL1, 2DL3, and 3DL1 that should all be licensed by their expression of HLA-C1, C2, and HLA-Bw4 (Supplemental Table S1). Donors 3 and 4 expressed KIR2DL1 but did not express its ligand HLA-C2; thus, the NK cells should not be licensed to kill (Supplemental Table S1). In the absence of HLA-C2 on Fadu, one might expect the KIR2DL1 licensed NK cells from Donors 1 and 2 to mediate more potent killing of Fadu than the NK cells from Donors 3 and 4. However, based on KIR/KIR-ligand genotype, one might also anticipate Donors 3 and 4 to induce Fadu killing similar to each other, but less than that seen by Donors 1 and 2. Yet, we observed that Donors 3 and 4 had different killing capacity, with Donor 3 showing Fadu killing similar to that of Donors 1 and 2. Thus, while some of the killing magnitude of Fadu cells by the mlNK cells from these four donors might in part be due to similarities or differences reflecting licensing and KIR inhibition determined by KIR and the KIR-ligand genotype of the donors and of the Fadu target cells, some of the observed killing magnitude must also reflect inherited, or acquired differences, other than those determined solely by inherited KIRs and KIR-ligands. Although KIR/KIR-ligand interaction has shown some potential to predict killing in naïve NK cells, this alone is not enough to predict the potency of mlNK cells. Therefore, we next looked at variability within mlNK products after memory induction.

### 3.3. NK Viability Remains High After Memory Protocol Despite Drop in Total Count

**Figure 2:**
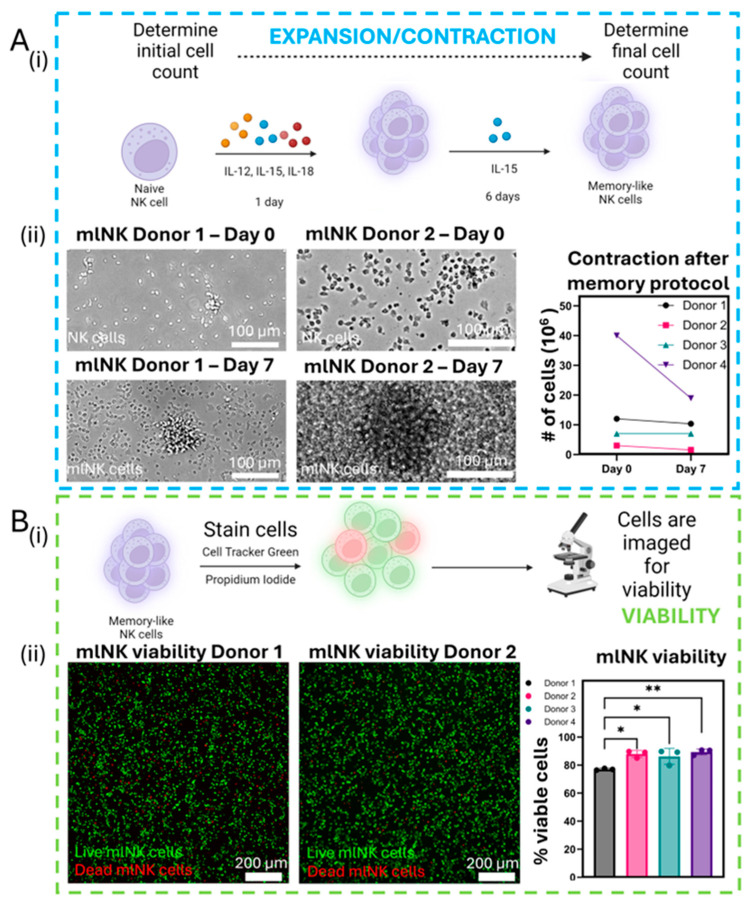
(**A**) (**i**) Graphical schematic showing mlNK cell generation and protocol followed for expansion/contraction. (**ii**) Brightfield mlNK images for Donors 1 and 2, accompanied by graph indicating contraction in total mlNK cell count across all four donors 7 days post-cytokine stimulation. (**B**) (**i**) Graphical schematic showing the staining protocol used to determine viability. (**ii**) Fluorescent images depicting viability of donor 1 and 2 mlNK cells. Live mlNK cells are stained in green with calcein and dead mlNK cells are stained in red with propidium iodide. Graph showcasing difference in mlNK cell viability across all donors. Graph shows average ± standard deviation. * and ** represent *p*-value < 0.05 and 0.01, respectively.

ACT often relies on the large-scale ex vivo expansion and activation of lymphocytes (e.g., T cells, NK cells) for future reinfusion into patients. Thus, we measured NK cell expansion over the course of the memory protocol (Figure 2A(i)). We observed that overall mlNK cell counts never exceeded the initial number that was seeded, i.e., no proliferation was seen (Figure 1A(ii)). Donor 4 saw the largest contraction, with a 52.6% decrease from 40 × 10^6^ to 18.95 × 10^6^ total NK cells. Other donors showed minimal to no difference in cell numbers. For example, Donor 1 only had a 13.7% reduction from 12 × 10^6^ total NK cells to 10.36 × 10^6^. Next, we assessed the viability of mlNK cells after 7 days of memory-like induction (Figure 2B(i)). The viability of mlNK cells was >75%, with results in the range of ~75–90% across donors (Figure 2B(ii) and Figure S4). Although cell count and viability are good indicators of cellular health, we did not observe a clear correlation between mlNK cell viability and their capacity to kill. Thus, we set out to analyze other relevant parameters that could inform the cell activation of NK cells in response to stimulus, such as cluster formation.

### 3.4. mlNK Cells Self-Organize into Clusters of Different Sizes

**Figure 3:**
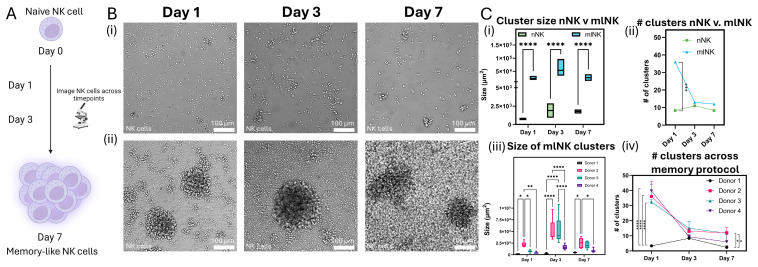
(**A**) Schematic overview of the clustering protocol used. (**B**) Brightfield images of (**i**) nNK and (**ii**) mlNK clusters on days 1, 3, and 7. (**C**) Comparison between nNK and mlNK cell (**i**) cluster size and (**ii**) number. Quantification of (**iii**) mlNK cluster size and (**iv**) number across donors. Graphs are box and whisker plots showing single-cell data. *, **, ***, and **** represent *p*-value < 0.05, <0.01, <0.001, and <0.0001, respectively.

The formation of clusters during NK cell growth and expansion is typically an indicator of activation [31]. Therefore, we sought to quantify cluster formation across multiple donors where mlNK cells were generated through the same transient exposure to cytokines. In this paper, we studied cluster formation seen over the course of the memory protocol (Figure 3A) for both naïve and mlNK cells. We collected microscopy images on three separate time points (1, 3, and 7 days after cytokine exposure) and evaluated changes in cluster formation (Figure 3B and Figure S5). There was a significant increase in the size of clusters observed when comparing naïve NK (nNK) cells to mlNK cells (Figure 3C(i)). The number of clusters identified was higher in the mlNK population after 1 day, suggesting increased cell-to-cell communication, IL-2 production, and thus activation [31]. However, by day 7, both populations had a similar number of clusters (Figure 3C(ii)). This was because mlNK clusters formed after day 1 and could have fused, resulting in fewer but larger clusters. In addition, the initial significant increase in the number of clusters in the mlNK population on day 1 could be associated with the rapid expansion phase following NK cell activation.

When comparing mlNK cells across donors, a general trend was observed where the size of the clusters began small on day 1 and grew larger by day 3 and finally contracted in size by day 7 (Figure 3B(ii)). On any given day, large variation across donors could be observed (Figure 3C). For example, day 3 shows a large spread of cluster sizes in Donor 2 and 3 compared to Donor 1 and 4. In addition to size, the number of clusters identified was assessed. In this case, 1 day after cytokine exposure we see a burst of clusters from the single-cell state observed on day 0. The number of clusters gradually decreases on day 3 and day 7. This trend is followed by all donors except Donor 1, where a majority of cells remained in a single-cell state on day 1 with a slight increase in number on day 3 followed by a decrease on the final time point (Figure 3C). Donor 4 is the most heterogeneous among the donors, with little increase in size and number of clusters. This suggests that NK cells from different donors undergo varying levels of cluster formation and activation over the course of the memory protocol despite being stimulated with the same cytokine cocktail. However, there was no clear correlation between cluster formation and cytotoxicity. Thus, we moved to study cell motility, which is relevant for mlNK cell tumor infiltration [32].

### 3.5. Single-Cell Motility Analysis Identifies Subsets of mlNK Cells with Differences in Their Migration Potential

**Figure 4:**
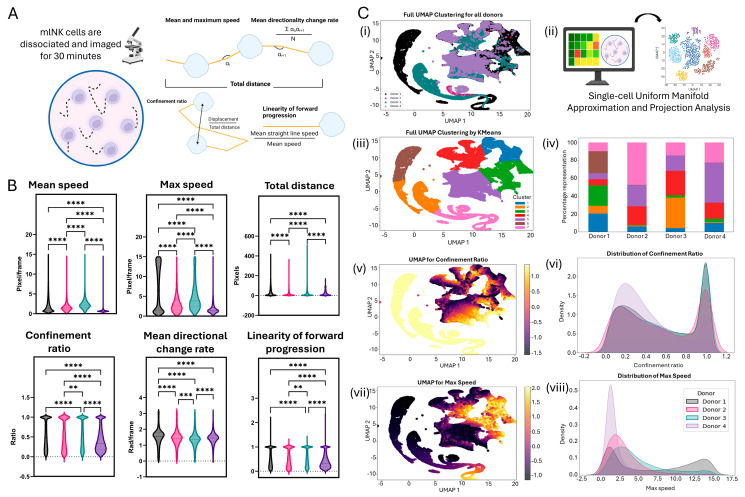
(**A**) Protocol used to evaluate mlNK motility and description of parameters analyzed. (**B**) Quantification of motility parameters—mean speed, max speed, total distance, confinement ratio, mean directional change rate, and linearity of forward progression—for each donor. (**C**) Uniform manifold approximation and projection analysis (UMAP). (**i**) Full UMAP clustering for all donors. (**ii**) Schematic of UMAP clustering. (**iii**) Full UMAP clustering by K-Means. (**iv**) Bar graph representation of cluster heterogeneity by donor. (**v**) UMAP for confinement ratio. (**vi**) Distribution of confinement ratio. (**vii**) UMAP for max speed. (**viii**) Distribution of max speed. Graphs are violin plots showing single-cell data. **, ***, and **** represent *p*-value < 0.01, <0.001, and <0.0001, respectively. Donor 1 shown in black, 2 in pink, 3 in green, and 4 in purple.

The motility of NK cells is crucial for their recruitment to tumor cells and infiltration within the tumor, enhancing cytotoxicity. Thus, we attempted to elucidate how variability in NK cells plays a role in the motility of mlNK cells. After a 7-day culture period, we collected mlNK cells, stained them with Calcein, and quantified their motility. The images were analyzed using the Trackmate plugin in Fiji to generate quantitative descriptors of NK cell motility (Figure 4A). We observed that for the mean speed of mlNK cells, Donors #1–4 have a similar spread, with most of the cells being below 5 pixels/frame. However, when we analyzed maximum speed, we observed a higher degree of heterogeneity across mlNK cells obtained from different donors. In this context, Donor 1 showed two distinct cell populations, with one cluster showing a five-fold increase in cell maximum speed (Cluster 1 < 5 pixels/frame vs. cluster 2 > 12 pixels/frame). Donors 2 and 3 on the other hand had higher median max speeds than Donors 1 and 4. In the total distance traveled, cells from each donor reached varying levels of distance traveled, with cells from Donor 3 reaching as high as 400 pixels traveled and Donor 4 cells traveling less than 200 pixels (Figure 4B). The confinement ratio is defined as the ratio of displacement of a cell to the total distance of path traveled. It is often used as a measure to define the straightness of a path, with 0 representing no net displacement, where the cell returns to its starting position, and 1 being a perfect straight line [33]. The linearity of forward progression refers to the ratio between mean straight-line speed and mean speed. It is another measure of the linearity of path traveled by a cell, wherein a value of 0 indicates a condensed (shorter net displacement) and random path and 1 indicates a completely straight line. For all donors, we saw the emergence of two populations of cells, one with a ratio tending to 0 and the other with a ratio closer to 1. Interestingly, Donor 4 showed a different distribution, with a larger population of cells having a linearity of forward progression of <0.5. The mean directionality change rate is defined as an average angle of deviation during a path followed by a cell. mlNK cells from each donor followed a similar trajectory, with the median number of changes being between 1 and 2 radians/frame (Figure 4B). This indicates a consistent slight course correction as opposed to a traveling in a perfectly straight line or major shifts in direction of path. It is to be noted that although there are some similar trends across donors for parameters of motility analysis, there are still significant differences across all donors, showcasing differences within a population as well as on a single-cell basis. Overall, Donor 4 shows an overall decreased and less energetic phenotype, as indicated by low speeds and distance traveled as well as path descriptors compared to other donors.

We performed a uniform manifold approximation and projection (UMAP) analysis to visualize heterogeneity in the motility dataset (Figure 4C(ii) and Figure S6). Additionally, kMeans clustering was used to quantify the cluster representation for each donor (Figure 4C(iii)). Donors 2 and 4 were predominantly represented by clusters 5 and 7, whereas Donor 1 had a more even distribution, while Donor 3 was predominated by cluster 2 (Figure 4C(iv)). This highlights how the motility of mlNK cells is variable across different mlNK products. In addition to inter-donor heterogeneity, UMAPs also allow the analysis of intra-donor heterogeneity. UMAP allows us to better understand parameters that may contribute to the donor’s heterogenous response compared to other donors. We examined parameter expression distribution within donors for all parameters (Figure S7). The UMAPs show that a cluster of cells with a high expression of confinement ratio is present on the bottom and left side for each donor. Similarly, the UMAP for maximum speed is heavily expressed in the upper right quadrant of the plots (Figure S7). For Donor 2, we can separate max speed cells (cluster to top right) and cells with a high linearity of forward progression (cluster to bottom right; Figure S7). In summary, the UMAP revealed the existence of multiple clusters of cells based on differences in their motility. Overall, there is a correlation between mlNK motility and cytotoxicity, especially with Donor 4, where we see indications of a less energetic migratory state.

### 3.6. Mitochondrial Architecture Can Be an Indicator of Metabolic Activity of mlNK Cells

**Figure 5:**
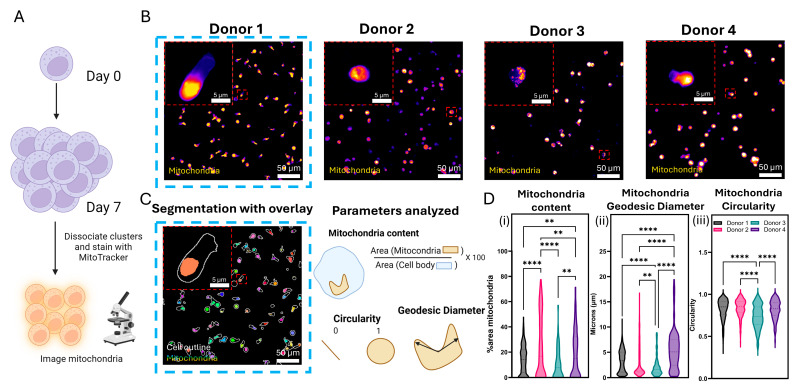
(**A**) Schematic overview of protocol used to analyze mitochondria content and geometry in mlNK cells. (**B**) Fluorescent images of mitochondria in mlNK cells obtained from different donors. (**C**) Images showing cell segmentation and parameters used to study mitochondria (mitochondria content, geodesic diameter, and circularity). Left panel includes mitochondria with surrounding cell body in white overlay. (**D**) Single-cell quantification of (**i**) mitochondrial content, (**ii**) mitochondrial geodesic diameter, and (**iii**) mitochondrial circularity. Graphs are violin plots showing single-cell data. ** and **** represent *p*-value < 0.01 and <0.0001, respectively.

Although activation and motility are good markers of cellular health, recent studies have suggested that NK cell metabolism plays an important role in downstream effector functions, with mitochondria having a central role [34]. Previous studies have shown that preactivating NK cells with a cytokine cocktail (IL-12, IL-15, and IL-18 for 18 h) resulted in cells exhibiting higher rates of glycolysis and identified mitochondrial polarization as a key factor contributing to NK cell activation and function [35]. Therefore, we looked at the mitochondrial size and structure of mlNK cells to determine metabolic fitness post memory induction for each donor. mlNK were obtained as previously described, their mitochondria stained and imaged using a 40× objective (Figure 5A) after memory induction. Fluorescence images (Figure 5B) were analyzed using MorphoLibJ in FIJI to quantify mitochondrial mass, geodesic diameter, and circularity (Figure 5C). Mitochondrial content was calculated by generating an ROI (region of interest) for the cell body and then calculating the percent area occupied by mitochondrial, providing a quantitative analysis of the mitochondria density on single-cell level. We saw significant variation across donors in mitochondrial content. A majority of Donor 1 mlNK cells had mitochondrial content within 10–30%, with very few cells reaching the 50% mark. On the other hand, Donor 2 mlNK cells had a larger spread of mitochondrial content (Figure 5D). Two distinctive populations of less than 20% and greater than 60% are observed in Donor 2, suggesting a split between cells undergoing low levels and high levels of energy-intensive processes, respectively, which are compatible with previous studies [36]. Donor 3’s mlNK cells were similar to Donor 1, with a maximum mitochondrial content of around 60%, with a majority of cells being less than 20%. Donor 4’s mlNK cells had mitochondrial content that demonstrated separation of two populations around the 10% and 30% mark, respectively (Figure 5D).

The geodesic diameter refers to the largest geodesic distance between two points in a defined region. Geodesic distance is the length of the shortest path joining two points within a region [37]. These parameters are often used as a tool to measure mitochondria length and network integrity status [38]. For example, cells with shorter geodesic diameter (short and fragmented mitochondria) have a lower capacity for oxidative phosphorylation (OXPHOS) in favor of heightened anaerobic metabolism [39]. Geodesic diameter values differ significantly between donors. Donor 1 has two populations of cells with geodesic diameter <2 microns and the other between the range of 5–10 microns (Figure 5D). The most elongated mitochondria have a geodesic diameter of around 12 microns. In contrast, the majority of Donor 2’s mlNK cells grouped together in a defined cluster with relatively short mitochondria (geodesic diameter less than 3 microns), with a few cells with mitochondria that were longer. Donor 3’s mlNK cells showed a similar shape, with the majority of cells consisting of a shorter geodesic diameter, with the most elongated mitochondria still being less than 10 microns. Finally, Donor 4’s mlNK cells had an even spread of mitochondrial in the range of 0–10 microns as well as having the most elongated mitochondria of nearly 20 microns (Figure 5D).

Like the geodesic diameter, circularity is another shape descriptor for mitochondrial network analysis. Circularity is defined by the ratio of area over the square of the perimeter of a given region. Circularity values of 1 are rounded in shape and a value of 0 is straight line [37]. The circularity quantification shows us that Donor 3’s mitochondria have a statistically significant difference compared to the other three donors. This difference is a shift towards a median value of 0.7, indicating a more elongated morphology compared to mitochondria in other donors. Most mitochondria from Donors 1, 2, and 4 had a circularity ratio closer to 1, with medians hovering around the 0.85 value (Figure 5D). Therefore, the circularity analysis depicts rounded and punctate mitochondria in these three donors. Overall, we observed a large variation in mitochondrial size and structure across the mlNK cells that were evaluated and no real correlation to trends observed in their killing potential. Altered mitochondrial structure and function can impair overall cellular metabolism, which, in turn, can derail the NK cell effector function [40]. Therefore, we decided to investigate changes in the expression of genes associated with glucose metabolism across all donors.

### 3.7. Gene Expression Analysis Suggests Disruption of Glycolytic Flux in mlNK Cells Could Affect Cytotoxicity

**Figure 6:**
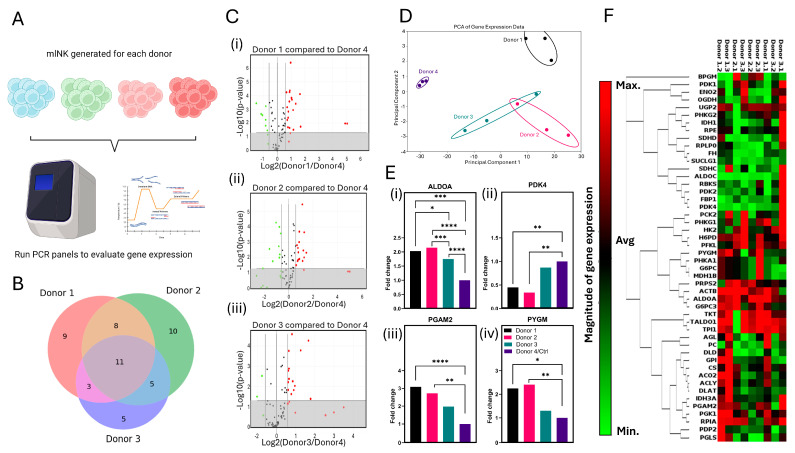
(**A**) Schematic overview of protocol used to analyze gene expression analysis. (**B**) Venn diagram depicting significantly dysregulated genes between Donors 1, 2, and 3 when compared to Donor 4. (**C**) Volcano plots depicting fold change in gene expression between (**i**) Donor 1 and Donor 4, (**ii**) Donor 2 and Donor 4, and (**iii**) Donor 3 and Donor 4. (**D**) PCA plot showcasing cluster separation between donors. (**E**) Graphical representation of fold change in (**i**) ALDOA, (**ii**) PDK4, (**iii**) PGAM2, and (**iv**) PYGM. (**F**) Clustergram showing magnitude of glucose metabolism gene expression for all donors. *, **, ***, and **** represent *p*-value < 0.05, <0.01, <0.001, and <0.0001, respectively.

Cellular metabolism has been shown to drive NK cell effector functions and especially anti-tumor effector functions [41]. In this study, we ran a gene expression analysis of mlNK cells from each donor to determine variability in their glucose metabolism (Figure 6A). For the analysis, Donors #1–3 were normalized to Donor #4, as it exhibited the most distinct response from previous experimentation described above. Of the genes analyzed, 11 were commonly dysregulated in all three donors, 16 were dysregulated in at least two donors, and 24 were only dysregulated in one donor (Figure 6B,C). A full list of genes in each of these categories is made available (Figure S8B). The top three pathways affected by the dysregulation of these genes were the Generation of Precursor Metabolites and Energy, Carbohydrate Metabolic Process, and Small Molecule Metabolic Process (Figure S8A). A principal component analysis (PCA) was run for all donors to evaluate overall heterogeneity between donors, with Donor 4 being the most separated from the others. Donors 2 and 3 have overlapping clusters, indicating their similarity in overall gene expression profiles (Figure 6D). It is important to note that the Donor 3 cluster is the one closest to the Donor 4 cluster, and implies that of the donors that are relatively similar (Donors 1, 2, and 3), Donor 3 is the closest to Donor 4, indicating similarity in fold change in metabolic gene expression. A few significantly dysregulated genes were selected to focus on and emphasize this pattern. The first was Aldolase A (*ALDOA*), a gene that plays a role in glycolysis and the maintenance of glucose homeostasis by facilitating the production of pyruvate [42,43]. Next, we looked at pyruvate dehydrogenase lipoamide kinase isozyme 4 (*PDK4*), a gene responsible for the inhibition of the pyruvate dehydrogenase (*PDH*) complex that results in a reduction in mitochondrial pyruvate flux, a decrease in OXPHOS, and increase in glycolysis [44,45]. Phosphoglycerate mutase 2 (*PGAM2*), a gene that aids in catalyzing the reversible conversion of 3-phosphoglycerate (3-PGA) to 2-phosphoglycerate (2-PGA) during glycolysis allowing for the production of pyruvate and the continuation of glycolysis, was also selected [46]. Finally, glycogen phosphorylase (*PYGM*), a gene that encodes enzymes involved in the breakdown of glycogen, was also selected for analysis [47,48]. For each gene, we observed that the fold change in Donor 3 was the closest to Donor 4, whereas while the fold change in Donors 1 and 2 were similar to each other, they were significantly different to Donor 4 (Figure 6E), suggesting the metabolic activity of Donor 3 was closest to that of Donor 4. Full clustergram shown in Figure 6F and Figure S9 highlights fold change across entire panel tested. These trends are consistent with the findings from the PCA plot and other readouts described such as cytotoxicity. As seen, each of these genes is expressed at a much different rate (ALDOA, PGAM2, and PYGM lower and PDK4 higher) in Donor 4 compared to the other donors, indicating a disruption of the glycolytic pathway for energy generation. This change could be correlated to the killing capacity of mlNK products from each donor, suggesting that mlNK cells’ ability to undergo glycolysis may have some underlying effect on their cytotoxic potential. Moreover, we saw the same trend across donors for both gene expression associated with glycolysis and their killing potential.

To further evaluate the importance of metabolism and metabolic fitness, we sought to evaluate the impact of blocking OXPHOS and glycolysis in mlNK cells. It has been shown that blocking either of these pathways caused altered function in mouse NK cells [49]. Thus, we generated mlNK cells in the presence and absence of 50 mM 2-DG (2-Deoxy-D-glucose; glycolysis inhibitor) and 100 nM oligomycin (OXPHOS inhibitor) and evaluated mlNK cell performance across several readouts. Overall, we observed impaired mlNK cell function for both donors tested (Figures S10 and S11). There was a significant decrease in their cytotoxic potential, viability, motility, and change in mitochondria content and morphology. Therefore, this highlights the importance of unimpaired metabolism in mlNK cells.

## 4. Discussion

The development of novel ACT for cancer will ultimately improve patient outcomes. However, with allogeneic ACT such as mlNK cell therapies, donor-to-donor variability in the quality of the mlNK products is a great concern and could be a driving factor in inconsistent results. Although there is an overall increase in memory-like markers in the mlNK population compared to the nNK population, consistent with previous studies [20], there is a large amount of variation in expression level between donors (Figure S1). Therefore, understanding the functional and molecular changes in mlNK cells that arise due to this variability is crucial. In this context, we conducted a panel of assays to predict mlNK cell potency.

For this study, we chose to use a HNSCC cell line as a model since NK cells are known to infiltrate these tumors and there is a positive correlation between NK cell infiltration and HNSCC overall survival [16]. NK cells are also known to target virally infected tumor cells such as Human Papillomavirus (HPV)+ cancers, which is one of the main HNSCC risk factors [16]. To avoid confounding the results due to donor HPV status, we chose to study if mlNK were capable of targeting and killing HPV–HNSCC cancer cells and thus the HPV–Fadu cell line was selected [50]. Donor 4 showed a distinctive behavior compared with the other donors wherein a substantial number of tumor cells are still viable after treatment with mlNK cells.

The donor mlNK cell KIR genotype could influence the mlNK killing capabilities. Yet, the lack of killing between Donor 4 and Donor 3, and the similarity in killing of Donor 3 compared to Donors 1 and 2 cannot readily be explained by differences or similarities in KIR/KIR-ligand NK cell licensing or NK cell inhibition. Thus, further analyses for molecular factors to delineate more robust predictors of mlNK cell quality would be highly beneficial for optimizing donor choice for NK cell ACT [51,52].

Next, we looked at NK cell expansion/contraction and viability upon memory induction to determine if its role on NK cell fitness and function. Across all donors, contraction of the total NK cell pool was observed after cytokine preactivation, resulting in a smaller portion of longer-lived pool of mlNK cells [53]. The viability of mlNK cells between donors remains relatively consistent with each donor showing 80% viability, indicating that although donors started off at different cell counts, they all contracted to a viability standard acceptable for adoptive transfer into humans for clinical testing.

Activation and clustering of NK cells influences NK cell effector function [31]. First, we compared nNK cluster size and number to mlNK cells. We observed a significant increase in the size of clusters, while the number of clusters tended to remain similar by the end of the memory protocol. NK cells pretreated with cytokines have been shown to proliferate rapidly and increase cell–cell communication, production of IL-2, and activation [18]. This may explain differences between nNK cells and mlNK cells grown in an elevated concentration of cytokines. For mlNK product comparisons, a general trend was observed, wherein the size of clusters initially increased followed by contraction in size by the end of the memory protocol. These results are similar to those described in the formation of memory T cells, wherein there is a period of rapid expansion, followed by contraction resulting in memory cells [53]. The number of clusters were also analyzed across the 7-day period, and we saw significant differences over the window of analysis, especially in Donor 1. Therefore, although trends are similar, variability can affect the extent to which mlNK cells cluster and activate greatly.

Next, we investigated the motility of mlNK cells to determine if differences in activation could predict heterogeneity in mlNK cell movement. Across all the motility analyses, there were significant differences across donors. We saw large heterogeneity in the max speed quantification graph, especially with Donor 1 having two very distinct populations—one with a higher max speed and the other with a lower max speed. This indicates two phenotypes—a faster mlNK cell that behaves as a ‘burst’ and cells that gain speed gradually but do not reach high maxes. The single-cell classification of NK cells into sub-populations has been previously studied in terms of cytotoxicity, wherein small subclasses of cells showcase “serial killer” characteristics to kill target cells in a fast ‘burst’ [54]. Further studies are required to determine if this moving population of cells are responsible for the rapid killing as well. In other parameters analyzed, Donor 4 showed the most phenotypic variability. mlNK cells from Donor 4 had the lowest mean speeds, traveled the least, and exhibited the most condensed paths. Additionally, we performed a UMAP on single-cell motility data to visualize the presence or lack of parameters that might provide us with insights into the separation of donor clusters. For example, Donor 4 UMAPs for each parameter lack the ‘tail’ shaped cluster. This could be correlated with the different phenotype observed in Donor 4 but would require further analysis to validate.

Mitochondrial fitness can also greatly affect cytokine production and the efficiency of glycolysis and OXPHOS in NK cells [55]. Mitochondrial content has often been used as a surrogate for mitochondrial biogenesis [56]. A greater mitochondrial content has even been associated with increased bioenergetic advantage in memory T cells [57]. We observed variability in mitochondria content across four donors. However, the mitochondrial content does not always depict the full picture of metabolic fitness. Structure and shape are also important descriptors for performance. We observed that Donors 2 and 4 had the most elongated mitochondria of the donor pool, suggesting a larger fused network of mitochondria. Mitochondrial fusion is often associated with decreased mitophagy, promotes OXPHOS, and overall metabolic adaptation to an inflammatory microenvironment [34,58]. We also saw that most donors had overall more circular (punctate) or fragmented mitochondria. Mitochondrial fission often results in the formation of fragmented mitochondria. It is important in cellular division, separation of damaged mitochondria, and transport [34]. Therefore, mitochondrial shape analyses indicate both the fused and fragmented morphology of mitochondria. This inconsistency could be a result of how the geodesic diameter analysis was performed without taking the cell size into consideration. Larger cells can have a longer geodesic diameter but may still be rounded. A further in-depth shape descriptor analysis could be of interest in the future.

Impaired or altered mitochondria can be due to changes in cellular metabolism in the cell. Therefore, we conducted RT-qPCR to evaluate changes in glucose metabolism gene expression across donors. In this case, we used Donor 4 as a control and evaluated gene expression changes. Overall, we saw genes that were commonly dysregulated in all three donors, in at least two donors and only in a single donor as well. We ran a PCA to evaluate how donors are clustering to visualize proximity to one another and noticed that the Donor 4 cluster was isolated with the other nearest cluster belonging to Donor 3. As previously mentioned, we saw that genes dysregulated in Donor 4 are suggestive of a widespread disruption of the glycolytic pathway for cellular metabolism. This could be correlated to the lack of cytotoxicity exhibited by mlNK cells from Donor 4. Moreover, the trend for dysregulation of glycolytic flux mimics the pattern observed from the cytotoxicity (Donor 4 < Donor 3 < Donors 1 and 2). To further characterize the impact of cellular metabolism, we also conducted a test where we blocked glycolysis and OXPHOS during the memory protocol to evaluate the impact on mlNK cell performance. We observed a marked decline in mlNK cytotoxicity alongside other notable disruptions to viability, motility, and mitochondria. In previous studies conducted on NK cell metabolism, glycolysis and OXPHOS were both increased upon activation of NK cells. They showcased an increase in glycolysis mediated pathways that contributed to NK cell cytotoxicity such as expression of Fas Ligand and granzyme B secretion [59]. The significance of OXPHOS has also been widely reported for NK cell metabolism and function, with studies suggesting immune memory cells have more reliance on OXPHOS for survival. Our results align with this highlighting the importance of proper cellular metabolism for optimal mlNK function.

Overall, this study demonstrates that the quality of mlNK products plays a critical role in determining the molecular and functional differences between donors. It is important to note that donors were tested negative for common infectious diseases and were all over the age of 50 years in order to maintain the consistency of the donor pool (Table S2). We observed that Donor 4 was the most heterogenous in several readouts, such as cytotoxicity, motility, and gene expression analysis. We observed correlations between the glycolytic capacity of mlNK cells and functional readouts, such as cytotoxicity and motility analyses, highlighting a potential method of distinguishing mlNK cell products for optimization.

This proof-of-concept study highlights the importance of studying the phenotypic and molecular heterogeneity of mlNK products but also showcases the need for many orthogonal parameters that could elucidate reasons for heterogeneity. Phenotypic characteristics of NK cells could be used as key predictors of NK cell anti-tumor efficacy alone or in combination with more traditional molecular screenings, ultimately leading to quicker and more effective donor-patient matches for ACT. By evaluating these properties, we can refine selection criteria for optimal NK cell donors. Moreover, the incorporation of these parameters in cell manufacturing quality assurance and quality control (QA/QC) may improve the overall standard and consistency across mlNK products. This could not only improve patient outcomes but also ensures alignment with strict regulatory standards set for cell manufacturing processes. In addition, with repeated testing and outcome analysis, we may be able to leverage artificial intelligence to build machine learning models that can identify optimal donors based on several parameters.

Although this study includes characterization of phenotypic and molecular heterogeneity of mlNK product across donors, only four donors were included in the analysis. This limits the ability of the study to form strong and conclusive correlations between observed trends. A future study dedicated to larger scale in-depth characterization of donor heterogeneity could validate trends indicated in this proof-of-concept study. Future studies elucidating correlation between these readouts will provide a more holistic understanding of how these parameters could be used as predictors for mlNK fitness and performance. Moreover, in this study we did not include protein-level assays to evaluate changes seen in gene expression. Future studies could further characterize mlNK dysfunction by conducting protein level analyses and metabolic flux experiments to evaluate glucose transport in mitochondria and cells when one or more pathways are inhibited (ex. glycolysis). Future studies using in vivo models or 3D culture systems like organ-on-a-chip and organoids in more complex environments that consider the inclusion of tumor stroma, hypoxia, and nutrient deprivation, and the role of specific immunosuppressive metabolites, may be needed [60,61].

## 5. Conclusions

This study underscores the importance of understanding donor-specific variability in mlNK cell products developed for ACTs. By comparing mlNK cells from four different donors, we identified significant differences in cytotoxicity, motility, clustering behavior, mitochondrial structure, and gene expression related to metabolism. Notably, the donors that exhibited reduced functional performance also displayed a distinct molecular signature, including disrupted glycolytic gene expression. These findings suggest that metabolic fitness may serve as a key indicator of mlNK cell efficacy and could be used to predict therapeutic potential. Ultimately, integrating these insights into a robust donor selection framework could enhance the consistency and effectiveness of mlNK-based adoptive cell therapies for cancer treatment.

## Data Availability

Data needed to make conclusions are presented in the paper and/or the Supplemental Document.

## Supplementary Materials

The following supporting information can be downloaded at https://www.mdpi.com/article/10.3390/cancers17142288/s1, Figure S1: Flow cytometry performed on naïve NK (nNK) and memory-like NK (mlNK) cells. Figure S2: mlNK cytotoxicity against Fadu head and neck cancer cells; Table S1: KIR and HLA genotype influence on licensing and inhibition for each donor; Figure S3: Fadu cells express MHC-I on their surface; Figure S4: NK cell viability after memory protocol; Figure S5: NK cell clustering during memory protocol; Figure S6: UMAP and distribution graphs of each motility parameter analyzed; Figure S7: UMAP heatmap plots for each motility parameter for all four donors separately; Figure S8: Gene expression data showcasing pathway dysregulation and specific genes dysregulated per donor(s); Figure S9: Clustergram showing the magnitude of gene expression for each donor; Table S2: Donor information table. Figure S10: Characterization of mlNK cells generated in the presence or absence of 50 mM 2-DG for two donors. Figure S11: Characterization of mlNK cells generated in the presence or absence of 100 nM oligomycin for two donors.

## References

[1] Zhang Y., Zhang Z. (2020). The history and advances in cancer immunotherapy: Understanding the characteristics of tumor-infiltrating immune cells and their therapeutic implications. Cell. Mol. Immunol..

[2] Lin D., Shen Y., Liang T. (2023). Oncolytic virotherapy: Basic principles, recent advances and future directions. Signal Transduct. Target. Ther..

[3] Van Der Bruggen P., Traversari C., Chomez P., Lurquin C., De Plaen E., Van Den Eynde B., Knuth A., Boon T. (1991). A Gene Encoding an Antigen Recognized by Cytolytic T Lymphocytes on a Human Melanoma. Science.

[4] Kirkwood J.M., Strawderman M.H., Ernstoff M.S., Smith T.J., Borden E.C., Blum R.H. (1996). Interferon alfa-2b adjuvant therapy of high-risk resected cutaneous melanoma: The Eastern Cooperative Oncology Group Trial EST 1684. J. Clin. Oncol..

[5] Hodi F.S., O’Day S.J., McDermott D.F., Weber R.W., Sosman J.A., Haanen J.B., Gonzalez R., Robert C., Schadendorf D., Hassel J.C. (2010). Improved survival with ipilimumab in patients with metastatic melanoma. N. Engl. J. Med..

[6] Leach D.R., Krummel M.F., Allison J.P. (1996). Enhancement of antitumor immunity by CTLA-4 blockade. Science.

[7] Rosenberg S.A., Restifo N.P. (2015). Adoptive cell transfer as personalized immunotherapy for human cancer. Science.

[8] Depil S., Duchateau P., Grupp S.A., Mufti G., Poirot L. (2020). ‘Off-the-shelf’ allogeneic CAR T cells: Development and challenges. Nat. Rev. Drug Discov..

[9] Mehta R.S., Rezvani K. (2016). Can we make a better match or mismatch with KIR genotyping?. Hematology.

[10] Seiwert T.Y., Burtness B., Mehra R., Weiss J., Berger R., Eder J.P., Heath K., McClanahan T., Lunceford J., Gause C. (2016). Safety and clinical activity of pembrolizumab for treatment of recurrent or metastatic squamous cell carcinoma of the head and neck (KEYNOTE-012): An open-label, multicentre, phase 1b trial. Lancet Oncol..

[11] Mell L.K., Torres-Saavedra P.A., Wong S.J., Kish J.A., Chang S.S., Jordan R.C., Liu T., Truong M.T., Winquist E.W., Takiar V. (2024). Radiotherapy with cetuximab or durvalumab for locoregionally advanced head and neck cancer in patients with a contraindication to cisplatin (NRG-HN004): An open-label, multicentre, parallel-group, randomised, phase 2/3 trial. Lancet Oncol..

[12] Laskowski T.J., Biederstädt A., Rezvani K. (2022). Natural killer cells in antitumour adoptive cell immunotherapy. Nat. Rev. Cancer.

[13] Ayuso J.M., Truttschel R., Gong M.M., Humayun M., Virumbrales-Munoz M., Vitek R., Felder M., Gillies S.D., Sondel P., Wisinski K.B. (2019). Evaluating natural killer cell cytotoxicity against solid tumors using a microfluidic model. Oncoimmunology.

[14] Schantz S.P., Shillitoe E.J., Brown B., Campbell B. (1986). Natural killer cell activity and head and neck cancer: A clinical assessment. J. Natl. Cancer Inst..

[15] Schantz S.P., Campbell B.H., Guillamondegui O.M. (1986). Pharyngeal carcinoma and natural killer cell activity. Am. J. Surg..

[16] Charap A.J., Enokida T., Brody R., Sfakianos J., Miles B., Bhardwaj N., Horowitz A. (2020). Landscape of natural killer cell activity in head and neck squamous cell carcinoma. J. Immunother. Cancer.

[17] Berrien-Elliott M.M., Wagner J.A., Cashen A.F., Fehniger T.A. (2018). Memory-Like Natural Killer Cells. Blood.

[18] Pahl J.H.W., Cerwenka A., Ni J. (2018). Memory-Like NK cells: Remembering a previous activation by cytokines and NK cell receptors. Front. Immunol..

[19] Berrien-Elliott M.M., Cashen A.F., Cubitt C.C., Neal C.C., Wong P., Wagner J.A., Foster M., Schappe T., Desai S., McClain E. (2020). Multidimensional anal-yses of donor memory-like NK cells reveal new associations with response after adoptive immunotherapy for leu-kemia. Cancer Discov..

[20] Romee R., Rosario M., Berrien-Elliott M.M., Wagner J.A., Jewell B.A., Schappe T., Leong J.W., Abdel-Latif S., Schneider S.E., Willey S. (2016). Cytokine-induced memory-like natural killer cells exhibit enhanced responses against myeloid leukemia. Sci. Transl. Med..

[21] Erbe A.K., Wang W., Carmichael L., Kim K., Mendonça E.A., Song Y., Hess D., Reville P.K., London W.B., Naranjo A. (2018). Neuroblastoma Patients’ KIR and KIR-Ligand Genotypes Influence Clinical Outcome for Dinutuximab-based Immunotherapy: A Report from the Children’s Oncology Group. Clin. Cancer Res..

[22] Ayuso J.M., Farooqui M., Virumbrales-Muñoz M., Denecke K., Rehman S., Schmitz R., Guerrero J.F., Sanchez-De-Diego C., Campo S.A., Maly E.M. (2023). Microphysiological model reveals the promise of memory-like natural killer cell immunotherapy for HIV ± cancer. Nat. Commun..

[23] Tu M.M., Mahmoud A.B., Makrigiannis A.P. (2016). Licensed and unlicensed NK cells: Differential roles in cancer and viral control. Front. Immunol..

[24] Pende D., Falco M., Vitale M., Cantoni C., Vitale C., Munari E., Bertaina A., Moretta F., Del Zotto G., Pietra G. (2019). Killer Ig-like receptors (KIRs): Their role in NK cell modulation and developments leading to their clinical exploitation. Front. Immunol..

[25] Moesta A.K., Norman P.J., Yawata M., Yawata N., Gleimer M., Parham P. (2008). Synergistic Polymorphism at Two Positions Distal to the Ligand-Binding Site Makes KIR2DL2 a Stronger Receptor for HLA-C Than KIR2DL3. J. Immunol..

[26] Almeida C.R., Ashkenazi A., Shahaf G., Kaplan D., Davis D.M., Mehr R. (2011). Human NK cells differ more in their KIR2DL1-dependent thresholds for HLA-Cw6-mediated inhibition than in their maximal killing capacity. PLoS ONE.

[27] Litwin V., Gumperz J., Parham P., Phillips J.H., Lanier L.L. (1994). NKB1: A natural killer cell receptor involved in the recognition of polymorphic HLA-B molecules. J. Exp. Med..

[28] Yokoyama W.M., Kim S. (2006). Licensing of natural killer cells by self-major histocompatibility complex class I. Immunol. Rev..

[29] Jonsson A.H., Yokoyama W.M. (2009). Natural killer cell tolerance licensing and other mechanisms. Adv. Immunol..

[30] Kim S., Poursine-Laurent J., Truscott S.M., Lybarger L., Song Y.J., Yang L., French A.R., Sunwoo J.B., Lemieux S., Hansen T.H. (2005). Licensing of natural killer cells by host major histocompatibility complex class I molecules. Nature.

[31] Kim M., Kim T.J., Kim H.M., Doh J., Lee K.M. (2017). Multi-cellular natural killer (NK) cell clusters enhance NK cell activation through localizing IL-2 within the cluster. Sci. Rep..

[32] Palacios D., Majhi R.K., Szabo E.K., Clement D., Lachota M., Netskar H., Penna L., Krokeide S.Z., Vincenti M., Kveberg L. (2024). The G Protein–Coupled Receptor GPR56 Is an Inhibitory Checkpoint for NK Cell Migration. J. Immunol..

[33] Tinevez J.Y., Perry N., Schindelin J., Hoopes G.M., Reynolds G.D., Laplantine E., Bednarek S.Y., Shorte S.L., Eliceiri K.W. (2017). TrackMate: An open and extensible platform for single-particle tracking. Methods.

[34] Xie J.H., Li Y.Y., Jin J. (2020). The essential functions of mitochondrial dynamics in immune cells. Cell. Mol. Immunol..

[35] Surace L., Doisne J.-M., Escoll P., Marie S., Dardalhon V., Croft C., Thaller A., Topazio D., Sparaneo A., Cama A. (2021). Polarized mitochondria as guardians of NK cell fitness. Blood Adv..

[36] Chen W., Zhao H., Li Y. (2023). Mitochondrial dynamics in health and disease: Mechanisms and potential targets. Signal Transduct. Target. Ther..

[37] Legland D., Arganda-Carreras I., Andrey P. (2016). MorphoLibJ: Integrated library and plugins for mathematical morphology with ImageJ. Bioinformatics.

[38] Gemmink A., Daemen S., Wefers J., Hansen J., Moorsel Dvan Astuti P., Jorgensen J.A., Kornips E., Schaart G., Hoeks J., Schrauwen P. (2023). Twenty-four hour rhythmicity in mitochondrial network connectivity and mitochondrial respiration; a study in human skeletal muscle biopsies of young lean and older individuals with obesity. Mol. Metab..

[39] Zheng X., Qian Y., Fu B., Jiao D., Jiang Y., Chen P., Shen Y., Zhang H., Sun R., Tian Z. (2019). Mitochondrial fragmentation limits NK cell-based tumor immunosurveillance. Nat. Immunol..

[40] Haythorne E., Lloyd M., Walsby-Tickle J., Tarasov A.I., Sandbrink J., Portillo I., Exposito R.T., Sachse G., Cyranka M., Rohm M. (2022). Altered glycolysis triggers impaired mitochondrial metabolism and mTORC1 activation in diabetic β-cells. Nat. Commun..

[41] Poznanski S.M., Ashkar A.A. (2019). What defines NK cell functional fate: Phenotype or metabolism?. Front. Immunol..

[42] Chang Y.C., Yang Y.C., Tien C.P., Yang C.J., Hsiao M. (2018). Roles of Aldolase Family Genes in Human Cancers and Diseases. Trends Endocrinol. Metab..

[43] Sobanski T., Suraweera A., Burgess J.T., Richard I., Cheong C.M., Dave K., Rose M., Adams M.N., O’Byrne K.J., Richard D.J. (2023). The fructose-bisphosphate, Aldolase A (ALDOA), facilitates DNA-PKcs and ATM kinase activity to regulate DNA double-strand break repair. Sci. Rep..

[44] Dou X., Fu Q., Long Q., Liu S., Zou Y., Fu D., Xu Q., Jiang Z., Ren X., Zhang G. (2023). PDK4-dependent hypercatabolism and lactate production of senescent cells promotes cancer malignancy. Nat. Metab..

[45] Flora G.D., Nayak M.K., Ghatge M., Kumskova M., Patel R.B., Chauhan A.K. (2023). Mitochondrial pyruvate dehydrogenase kinases contribute to platelet function and thrombosis in mice by regulating aerobic glycolysis. Blood Adv..

[46] Wiśniewski J., Barciszewski J., Turlik J., Baran K., Duda P., Jaskolski M., Rakus D. (2022). High-Resolution Crystal Structure of Muscle Phosphoglycerate Mutase Provides Insight into Its Nuclear Import and Role. Int. J. Mol. Sci..

[47] Migocka-Patrzałek M., Elias M. (2021). Muscle glycogen phosphorylase and its functional partners in health and disease. Cells.

[48] PYGM Glycogen Phosphorylase, Muscle Associated—NIH Genetic Testing Registry (GTR)-NCBI. https://www.ncbi.nlm.nih.gov/gtr/genes/5837/.

[49] Keppel M.P., Saucier N., Mah A.Y., Vogel T.P., Cooper M.A. (2015). Activation-specific metabolic requirements for NK cell IFN-γ production. J. Immunol..

[50] Spiotto M.T., Taniguchi C.M., Klopp A.H., Colbert L.E., Lin S.H., Wang L., Frederick M.J., Osman A.A., Pickering C.R., Frank S.J. (2021). Biology of the radio- and chemo-responsiveness in HPV malignancies. Semin. Radiat. Oncol..

[51] Wang W., Erbe A.K., Alderson K.A., Phillips E., Gallenberger M., Gan J., Campana D., Hank J.A., Sondel P.M. (2016). Human NK cells maintain licensing status and are subject to killer immunoglobulin-like receptor (KIR) and KIR-ligand inhibition following ex vivo expansion. Cancer Immunol. Immunother. CII.

[52] Wang W., Erbe A.K., Desantes K.B., Sondel P.M. (2017). Donor selection for ex vivo-expanded natural killer cells as adoptive cancer immunotherapy. Future Oncol..

[53] O’Sullivan T.E., Sun J.C., Lanier L.L. (2015). Natural Killer Cell Memory. Immunity.

[54] Vanherberghen B., Olofsson P.E., Forslund E., Sternberg-Simon M., Khorshidi M.A., Pacouret S., Guldevall K., Enqvist M., Malmberg K.J., Mehr R. (2013). Classification of human natural killer cells based on migration behavior and cytotoxic response. Blood.

[55] O’Brien K.L., Finlay D.K. (2019). Immunometabolism and natural killer cell responses. Nat. Rev. Immunol..

[56] Lamb R., Bonuccelli G., Ozsvári B., Peiris-Pagès M., Fiorillo M., Smith D.L., Bevilacqua G., Mazzanti C.M., McDonnell L.A., Naccarato A.G. (2015). Mitochondrial mass, a new metabolic biomarker for stem-like cancer cells: Understanding WNT/FGF-driven anabolic signaling. Oncotarget.

[57] Van Der Windt G.J., O’Sullivan D., Everts B., Huang S.C.C., Buck M.D., Curtis J.D., Chang C.H., Smith A.M., Ai T., Faubert B. (2013). CD8 memory T cells have a bioenergetic advantage that underlies their rapid recall ability. Proc. Natl. Acad. Sci. USA.

[58] Duroux-Richard I., Roubert C., Ammari M., Présumey J., Grün J.R., Häupl T., Grützkau A., Lecellier C.H., Boitez V., Codogno P. (2016). miR-125b controls monocyte adaptation to inflammation through mitochondrial metabolism and dynamics. Blood.

[59] Wang Z., Guan D., Wang S., Chai L.Y.A., Xu S., Lam K.P. (2020). Glycolysis and Oxidative Phosphorylation Play Critical Roles in Natural Killer Cell Receptor-Mediated Natural Killer Cell Functions. Front. Immunol..

[60] Reed-McBain C.A., Turaga R.V., Zima S.R.T., Abizanda Campo S., Riendeau J., Contreras Guzman E., Juang T.D., Juang D.S., Hampton D.W., Skala M.C. (2023). Microfluidic device with reconfigurable spatial temporal gradients reveals plastic astrocyte response to stroke and reperfusion. Lab Chip.

[61] Reed-McBain C., Turaga R.V., Zima S.R.T., Patel J., Cunha A.W.F., Mixdorf J., Wehner L.E., Engle J.W., Hernandez R., Rehen S.K. (2025). Non-destructive luminescence and PET imaging to monitor tissue microenvironment in microphysiological systems during brain metastasis using dissociated cerebral organoids. Biofabrication.

