# Supplementary material for: Analysis of Phenotypic and Molecular Variability of Memory-like NK Cells for Cancer Adoptive Cell Therapy Screening
Source: Cancers (Basel). 2025 Jul 9;17(14):2288. doi: 10.3390/cancers17142288 (PMC12293829; doi:10.3390/cancers17142288)
Supplement: Supplementary file 1 [file cancers-17-02288-s001.zip › cancers-3729061-supplementary.pdf]

SUPPLEMENTAL DOCUMENT

ANALYSIS OF PHENOTYPIC AND MOLECULAR VARIABILITY OF MEMORY-LIKE NK CELLS FOR CANCER ADOPTIVE CELL THERAPY SCREENING

Rithvik V. Turaga[1,2,3], Seth R.T. Zima[1,2,3], Ella P. Peterson[1,2,3], Amy K. Erbe[3,4], Matthew H. Forsberg[5], Christian M. Capitini[3,5] Pippa F. Cosper[3,4] ,Paul M. Sondel[3,4,5], Jose M. Ayuso[1,2,3,#]

[1]Department of Dermatology, School of Medicine and Public Health, University of Wisconsin, 1 S Park Street, Madison, WI, USA

[2]Department of Biomedical Engineering, College of Engineering, University of Wisconsin, 1550 Engineering Dr, Madison, WI, USA

[3]UW Carbone Cancer Center, 600 Highland Avenue, Madison, WI 53792, USA

[4] Department of Human Oncology, University of Wisconsin School of Medicine and Public Health, Madison, WI 53792, USA

[5]Department of Pediatrics, University of Wisconsin School of Medicine and Public Health, Madison, WI 53792, USA

#Corresponding author: ayusodomingu@wisc.edu

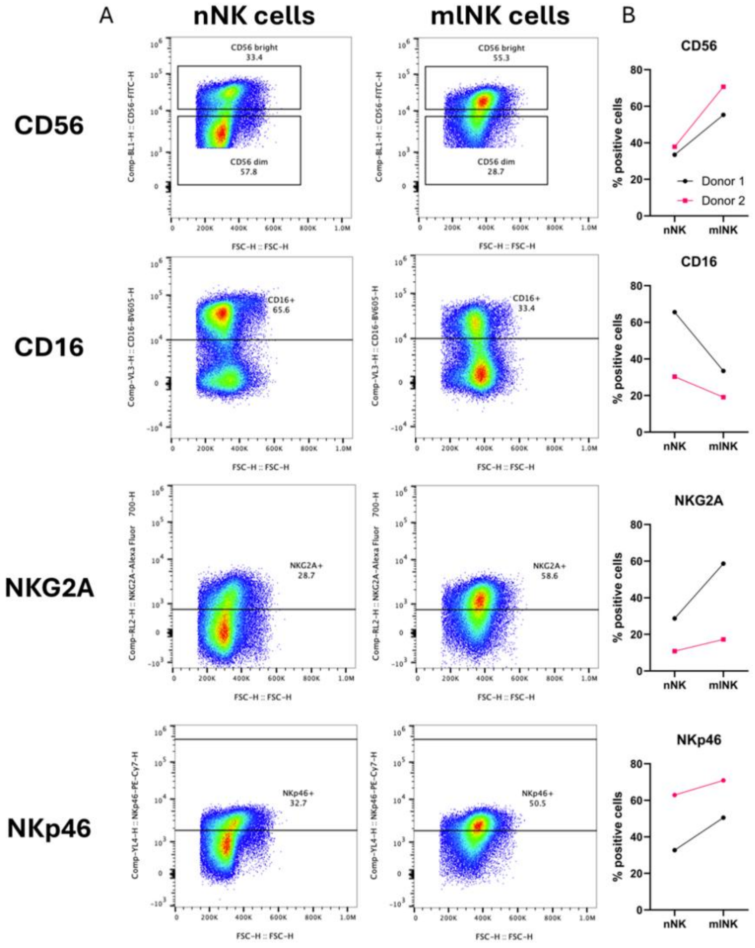

Supplemental Figure S1: Flow cytometry performed on Naïve NK (nNK) and memory-like NK (mlNK) cells. Flow plots for CD56, CD16, NKG2A and NKp46 on nNK cells (left) and memory-like NK cells (right). Graphs on the right are the aggregate of all tested donors for each readout comparing nNK to mlNK.

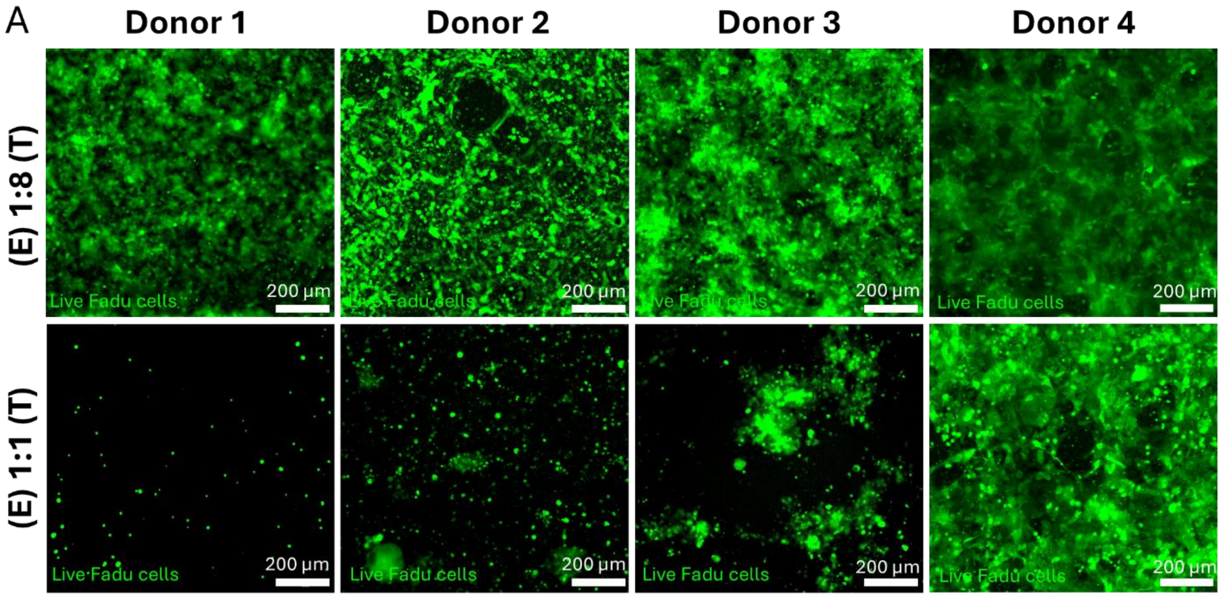

Supplemental Figure S2. mlNK cytotoxicity against Fadu target cells. Figure showcasing mlNK cytotoxicity for each donor at both effector to target ratios – 1:1 and 1:8.

	KIR Genotype				HLA Genotype			KIR/KIR-Ligand lIcensed?	KIR/Fadu-HLA Inhibited?
**mlNK donor**	**2DL1** (Ligand = C2)	**2DL2** (Ligand = C1)	**2DL3** (Ligand = C1)	**3DL1** (Ligand = Bw4)	**C1**	**C2**	**Bw4**		
Donor 1	+	-	+	+	+	+	+	2DL1, 2DL3, 3DL1: Yes	Yes (Not for KIR2DL1)
Donor 2	+	-	+	+	+	+	+	2DL1, 2DL3, 3DL1: Yes	Yes (Not for KIR2DL1)
Donor 3	+	+	+	+	+	-	+	2DL2/2DL3 and 3DL1: Yes KIR2DL1: No	Yes (Not for KIR2DL1)
Donor 4	+	-	+	+	+	-	+	2DL2/2DL3 and 3DL1: Yes KIR2DL1: No	Yes (Not for KIR2DL1)
**FADU**	N/A	N/A	N/A	N/A	**+**	**-**	**+**		

Supplemental Table S1. KIR and HLA genotype influence on licensing and inhibition for each donor. KIR2DL1 is a receptor for HLA-C2, KIRs 2DL2/2DL3 are receptors for HLA-C1, and KIR3DL1 is a receptor for HLA-Bw4. Fadu genotype as positive for HLA-C1 and HLA-Bw4; NK cells that express KIR2DL2, 2DL3 and/or 3DL1 can be inhibited by Fadu cells. Because Fadu cells lack HLA-C2, NK cells that express KIR2DL1 may not be inhibited by Fadu cells (unless other inhibitory markers on NK cells that can be encountered by Fadu cells, like KIRs 2DL2, 2DL3 or 3DL1, are co-expressed).

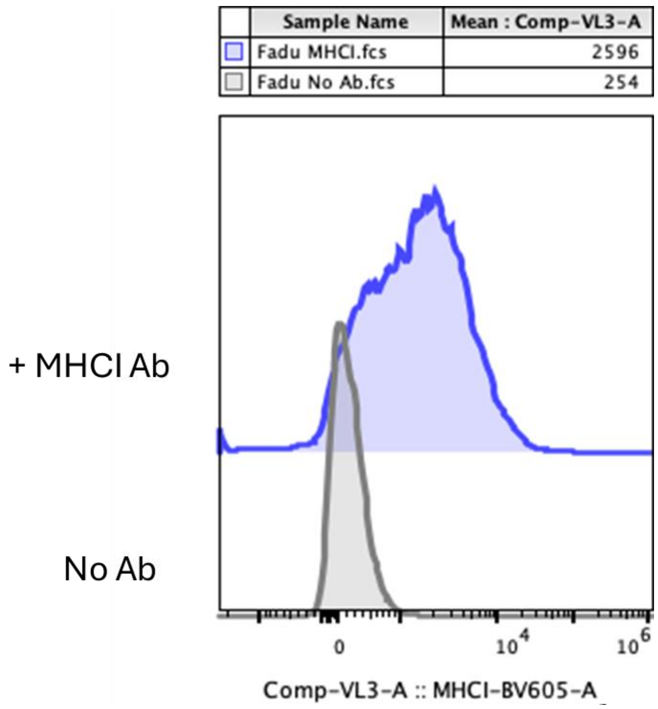

Supplemental Figure S3. Fadu cells express MHC-I on their surface. Fadu cells were incubated with HLA-A,B,C (clone W6.32) for 30 min. After cells were washed in flow buffer (PBS + 2%FBS), live cells (based on DAPI-negative) were assessed for MHC-I expression using an Attune NxT Flow Cytometer (ThermoFisher). Data were analyzed using FlowJo software.

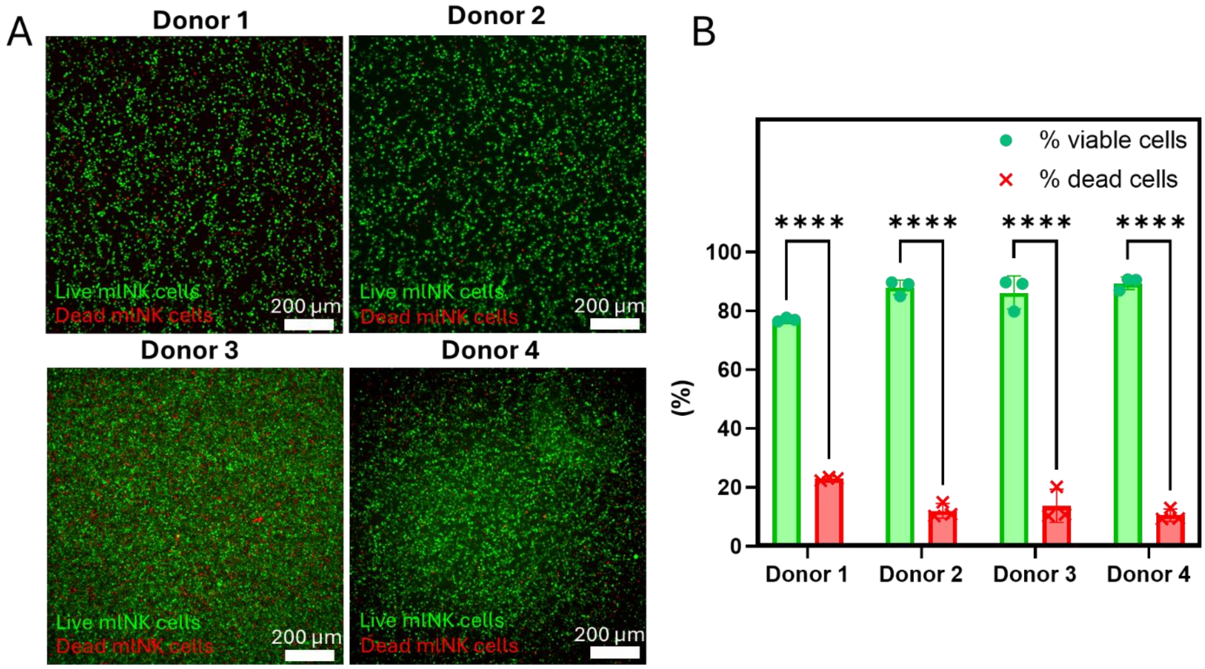

Supplemental Figure S4. NK cell viability after memory protocol. (A) Fluorescent microscopy images showing live cells stained with cell tracker green (green) and dead cells labeled with propidium iodide (red) for each donor after memory induction. (B) Quantification of percentage of viable and dead mlNK cells for each donor.

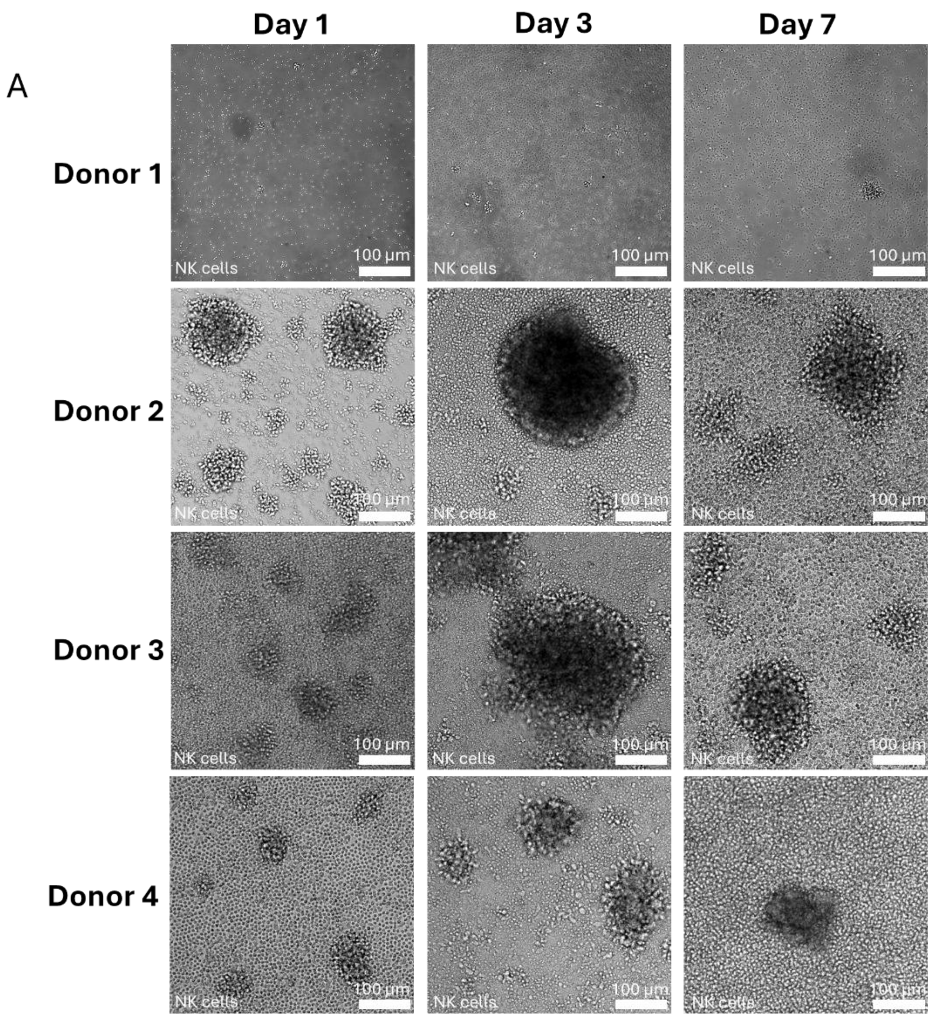

Supplemental Figure S5. NK cell clustering during memory protocol. (A) Brightfield images of cluster formation for each donor at three timepoints during the memory protocol.

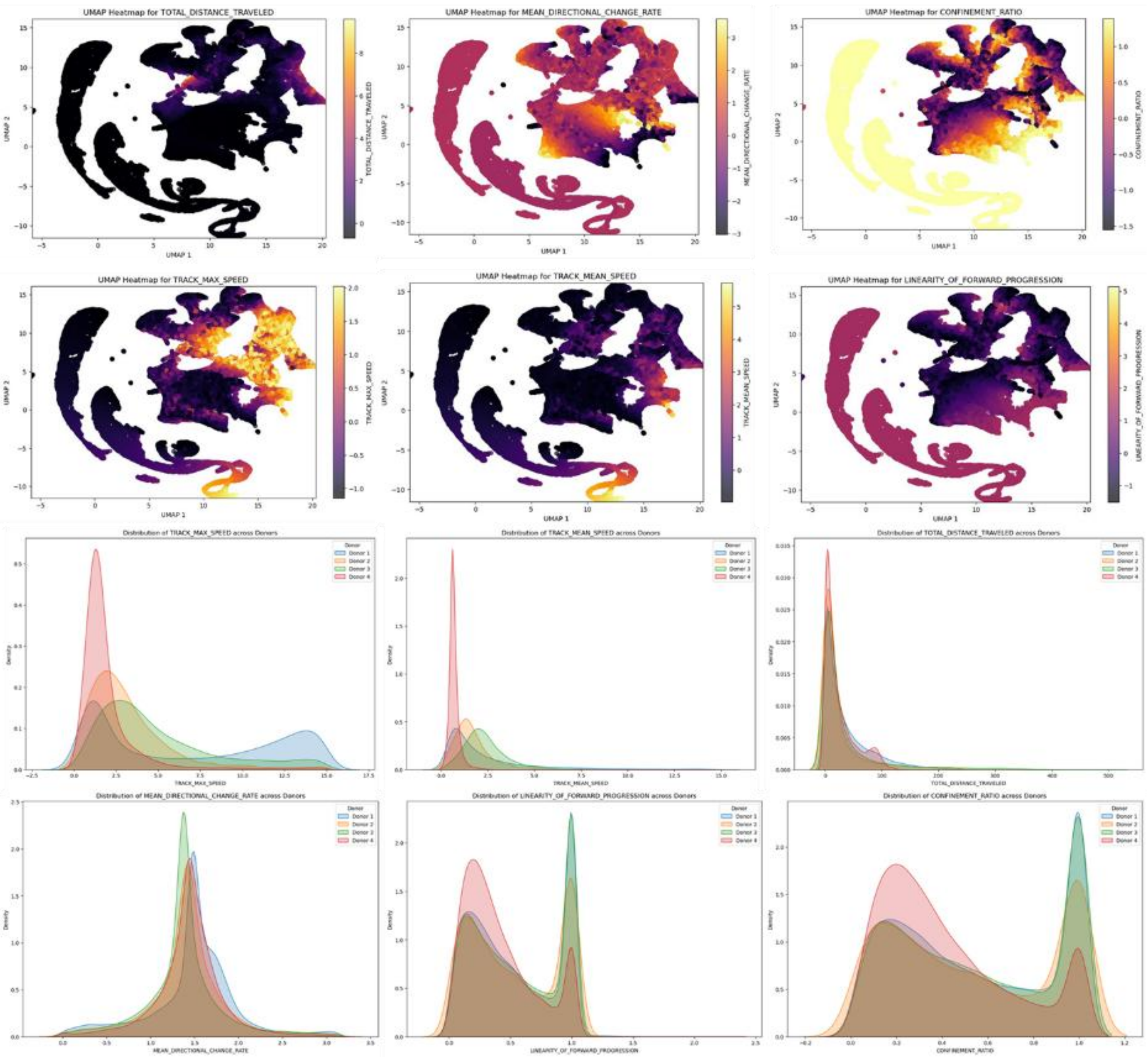

Supplemental Figure S6: UMAP and distribution graphs of each motility parameter analyzed.

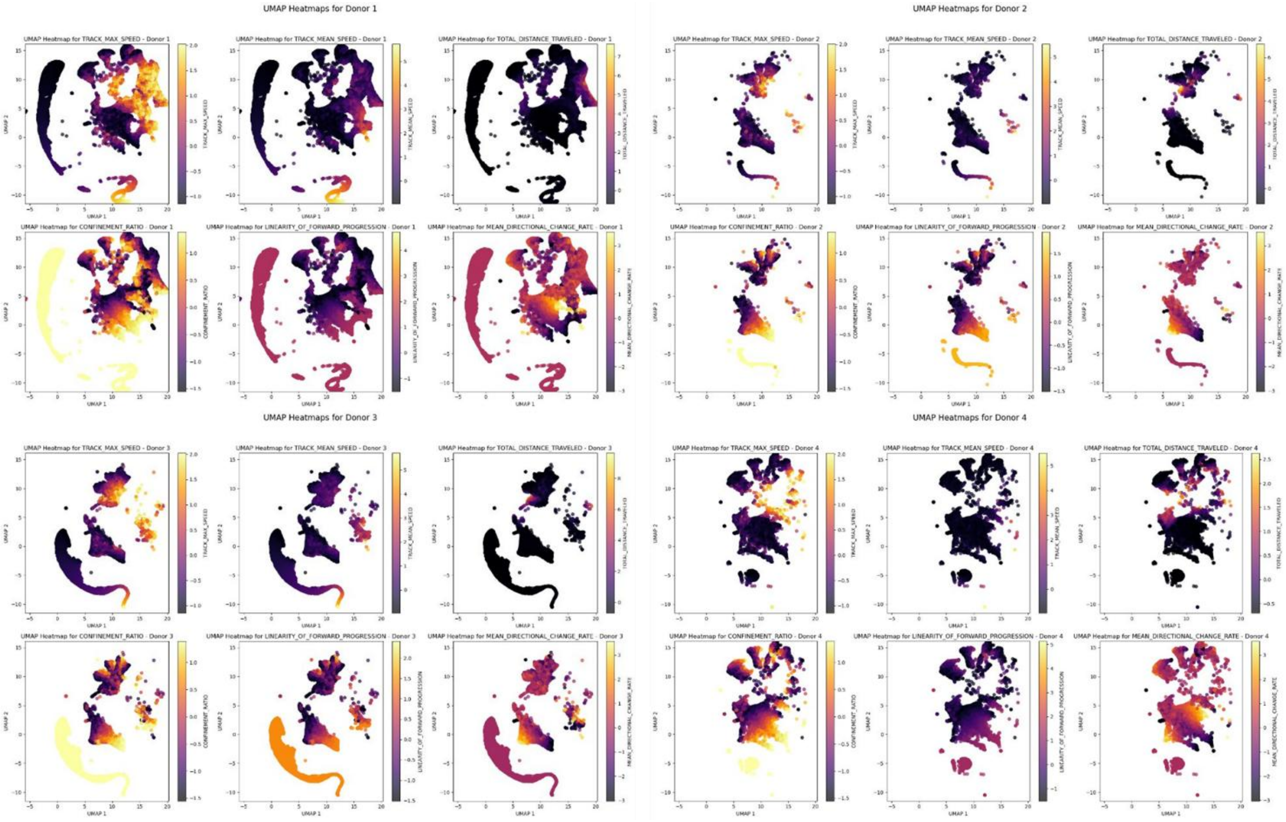

Supplemental Figure S7: UMAP heatmap plots for each motility parameter for all 4 donors separately.

A

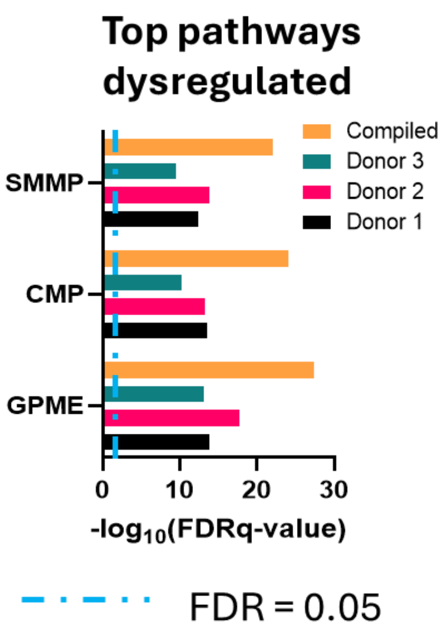

B

Donor 1	Donor 2	Donor 3	D1 & D2	D1 & D3	D2 & D3	D1 & D2 & D3
BPGM	BPGM	G6PC	BPGM	SUCLG1	PFKL	SUCLG1
G6PC	G6PC	PCK2	G6PC	RPIA	SUCLG1	GALM
FBP1	IDH1	FH	FBP1	PGK1	ALDOC	GSK3A
PGAM2	FH	PGAM2	PGAM2	GALM	GALM	G6PC
CS	FBP1	PFKL	G6PC3	IDH3A	HK2	ACLY
G6PC3	PGAM2	GSK3A	PYGM	GSK3A	GSK3A	ACTB
PYGM	G6PC3	ACLY	GSK3A	G6PC	G6PC	PHKG1
GSK3A	PYGM	ACTB	ACLY	ACLY	ACLY	PGAM2
ACLY	PFKL	ALDOA	ACO2	ACTB	FH	ALDOA
ACO2	TKT	SUCLG1	ACTB	PHKG1	ACTB	MDH1B
ACTB	GSK3A	PHKA1	ALDOA	PGAM2	PRPS2	H6PD
ALDOA	ACLY	HK2	PDK4	ALDOA	PHKG1	
PDK1	ACO2	PRPS2	SUCLG1	MDH1B	PGAM2	
DLAT	ACTB	AGL	MDH1B	H6PD	ALDOA	
PDK4	PHKG2	IDH3A	H6PD		MDH1B	
SUCLG1	ALDOA	MDH1B	GALM		H6PD	
ENO1	RPE	H6PD	SDHD			
IDH3A	PDK4	ALDOC	PDK2			
PDP2	SUCLG1	ENO2	PHKG1			
MDH1B	PGLS	RPIA				
H6PD	HK2	PGK1				
GPI	UGP2	GALM				
PC	PRPS2	PHKG1				
PGK1	SDHC	OGDH				
RPIA	TALDO1					
GALM	MDH1B					
SDHD	H6PD					
DLD	ALDOC					
PDK2	GALM					
PHKG1	SDHD					
RBKS	PDK2					
	TPI1					
	PHKG1					
	RPLP0					

Supplemental Figure S8: Gene expression data showcasing pathway dysregulation and specific genes dysregulated per donor(s). (A) Bar graph depicting top three pathways predicted to be most dysregulated by investigation of ontology gene sets. From top to bottom pathways are – Small Molecule Metabolic Process. Carbohydrate Metabolic Process, Generation of Precursor Metabolites and Energy. (B) Table showcasing genes dysregulated in single donor, at least 2 donors or all 3 donors. Table corresponds to Venn diagram in Figure 6B.

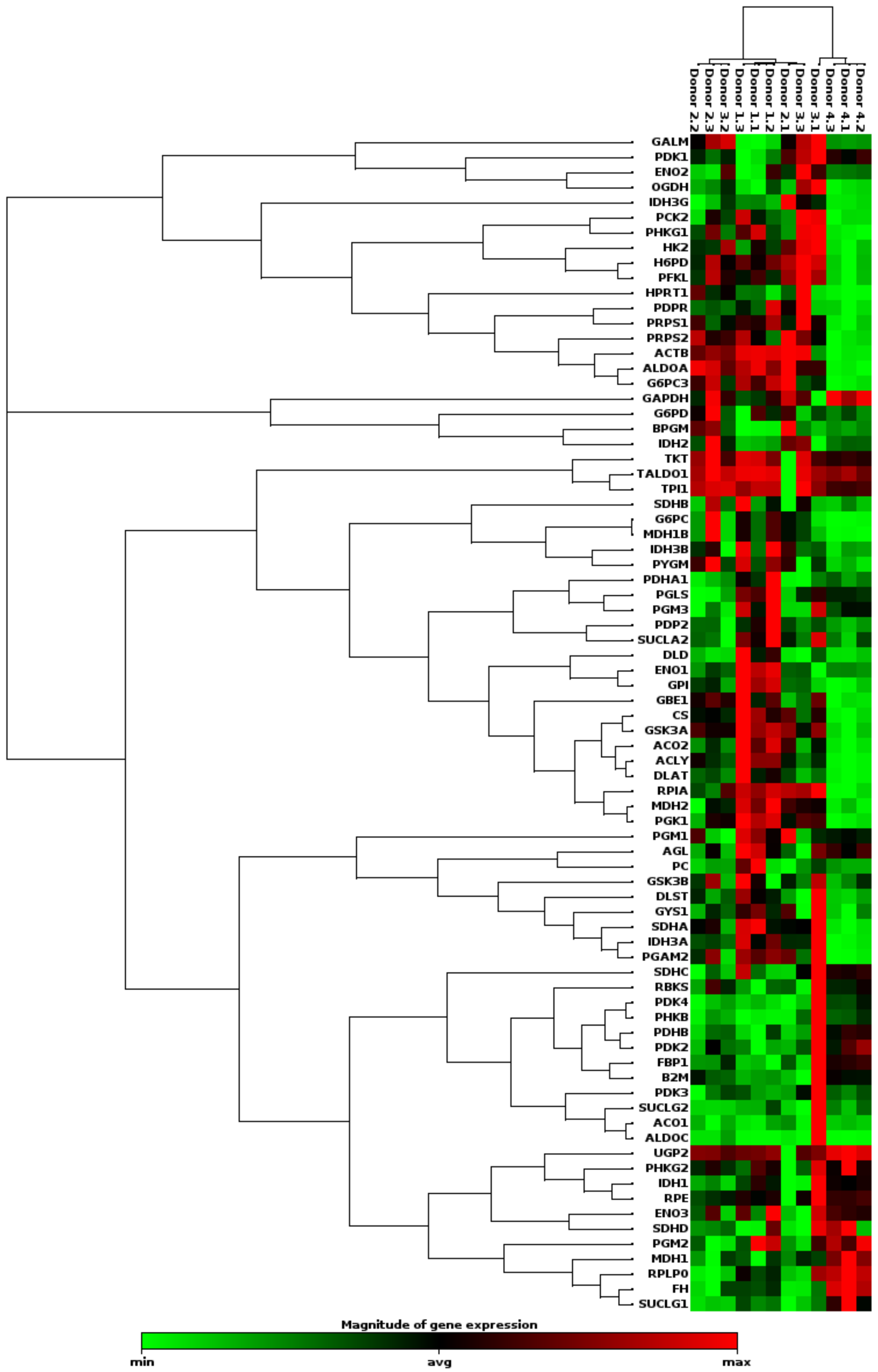

Supplemental Figure S9: Clustergram showing magnitude of gene expression for each donor.

Donor #	Age	Infectious Disease Results
1	53	Negative
2	62	Negative
3	67	Negative
4	67	Negative

Supplemental Table S2: Donor information table

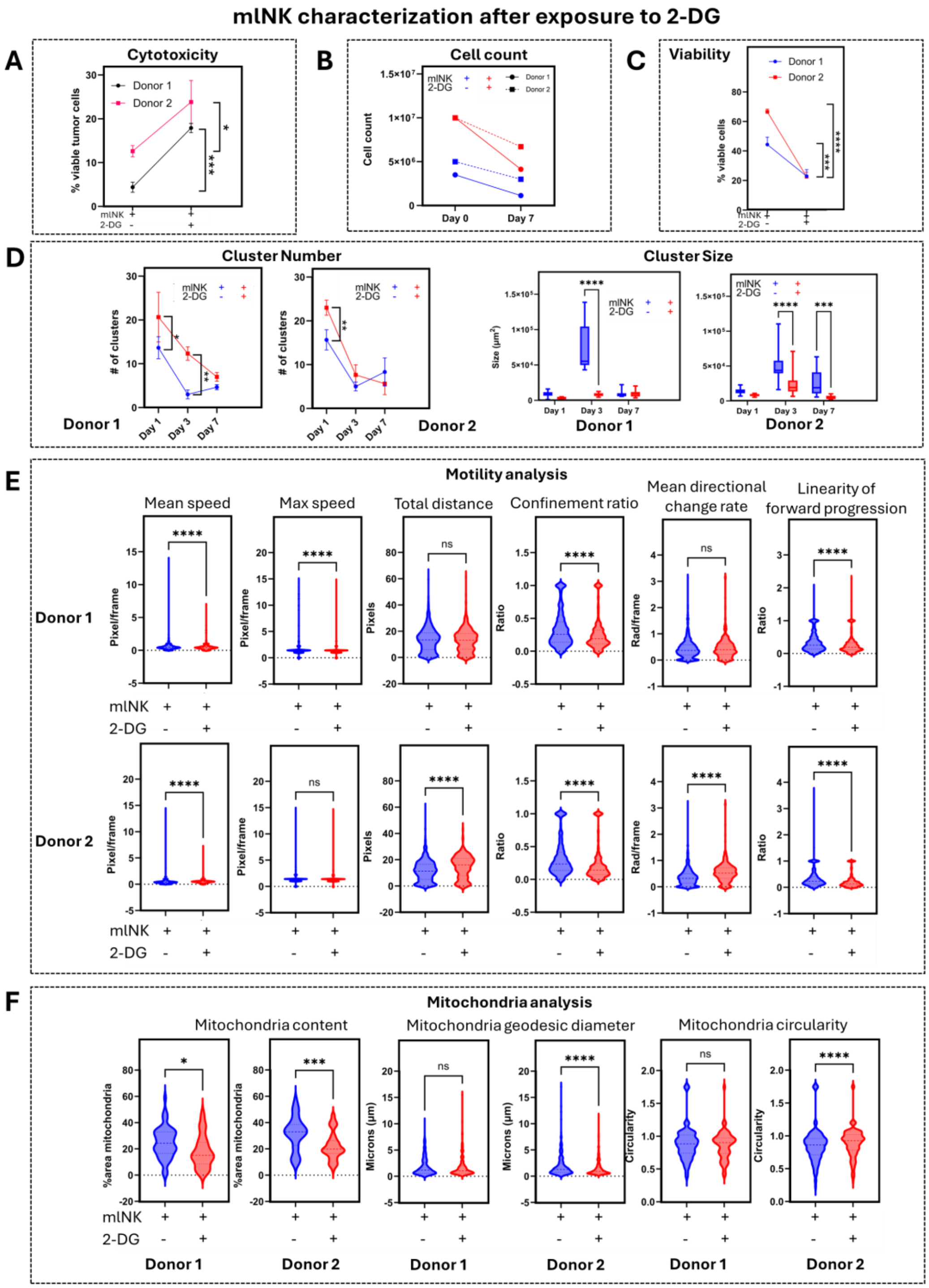

Supplemental Figure S10: Characterization of mlNK cells generated in the presence and absence of 50mM 2-DG for two donors. (A) Cytotoxicity of mlNK cells against Fadu tumor. (B) Contraction of mlNK cell population after memory protocol. (C) Viability of mlNK cells after memory protocol. (D) Cluster number and size after memory protocol. (E) Motility analysis across several parameters of mlNK cells. (F) Mitochondria morphology analysis of mlNK cells.

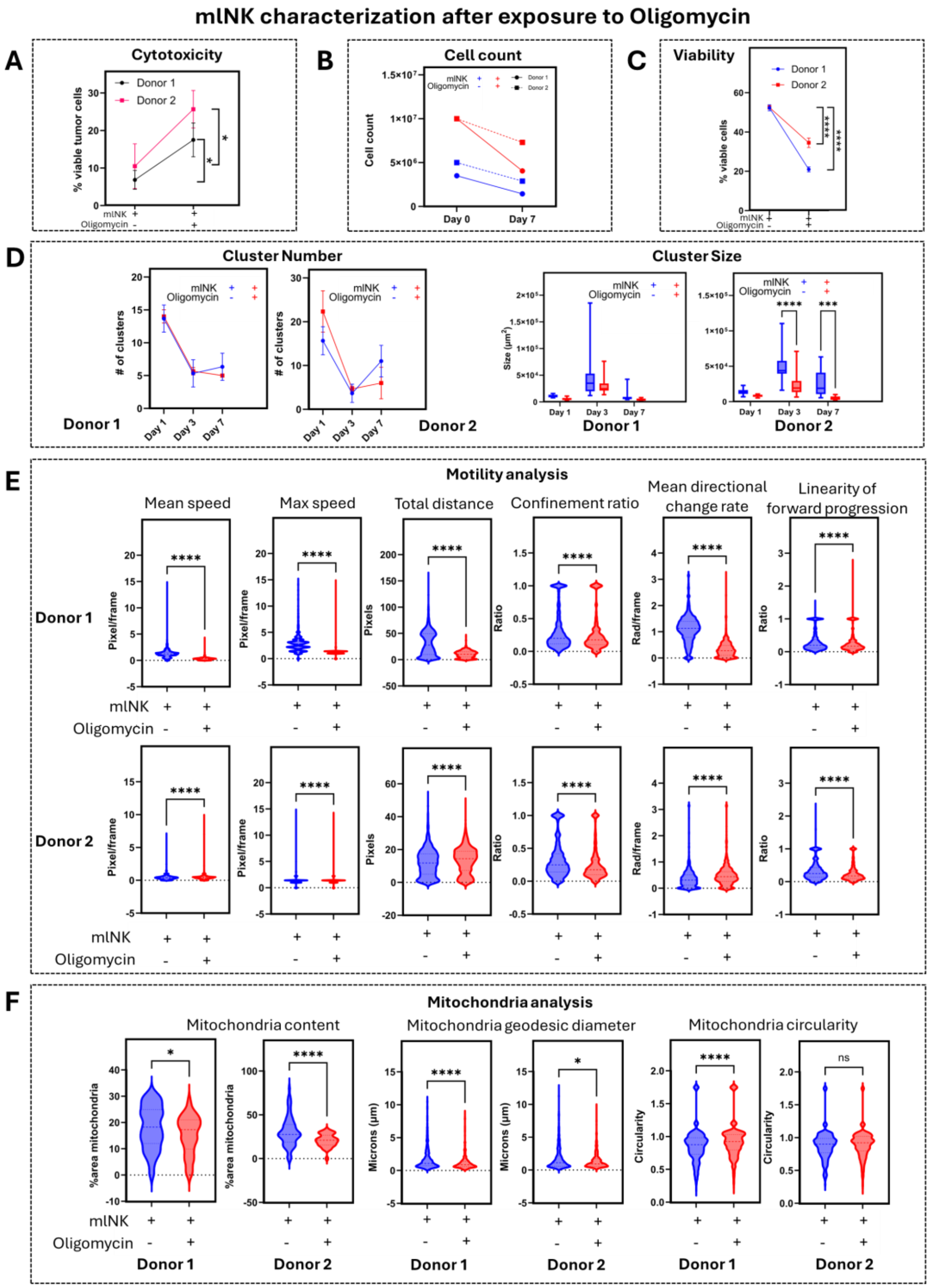

Supplemental Figure S11: Supplemental Figure 10: Characterization of mlNK cells generated in the presence and absence of 100nM oligomycin for two donors. (A) Cytotoxicity of mlNK cells against Fadu tumor. (B) Contraction of mlNK cell population after memory protocol. (C) Viability of mlNK cells after memory protocol. (D) Cluster number and size after memory protocol. (E) Motility analysis across several parameters of mlNK cells. (F) Mitochondria morphology analysis of mlNK cells.
