# Supplementary figures and images for: Analysis of Phenotypic and Molecular Variability of Memory-like NK Cells for Cancer Adoptive Cell Therapy Screening
Source: Cancers (Basel). 2025 Jul 9;17(14):2288. doi: 10.3390/cancers17142288 (PMC12293829; doi:10.3390/cancers17142288)

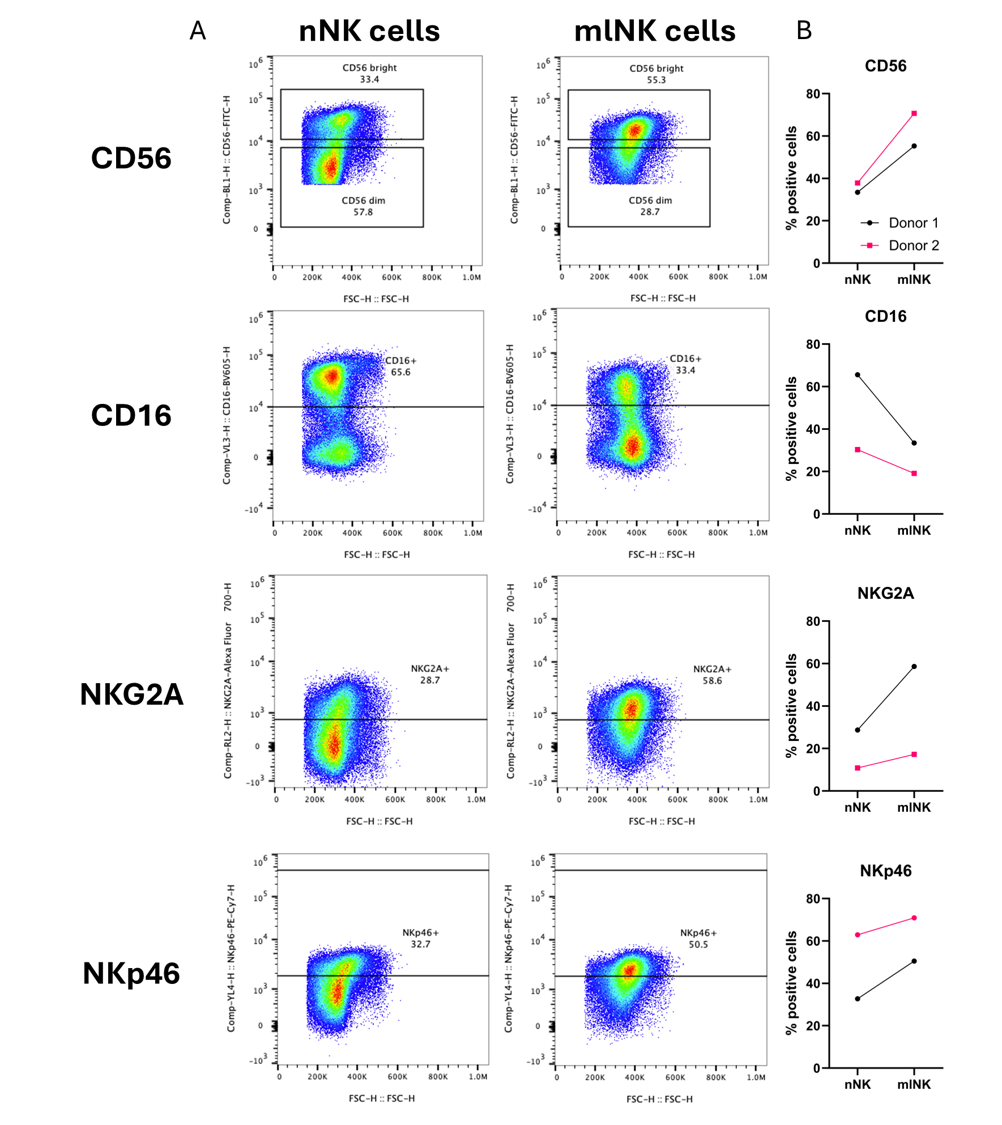

Supplement: Supplementary file 1 [file cancers-17-02288-s001.zip › Supplementary Figure S1.tif]

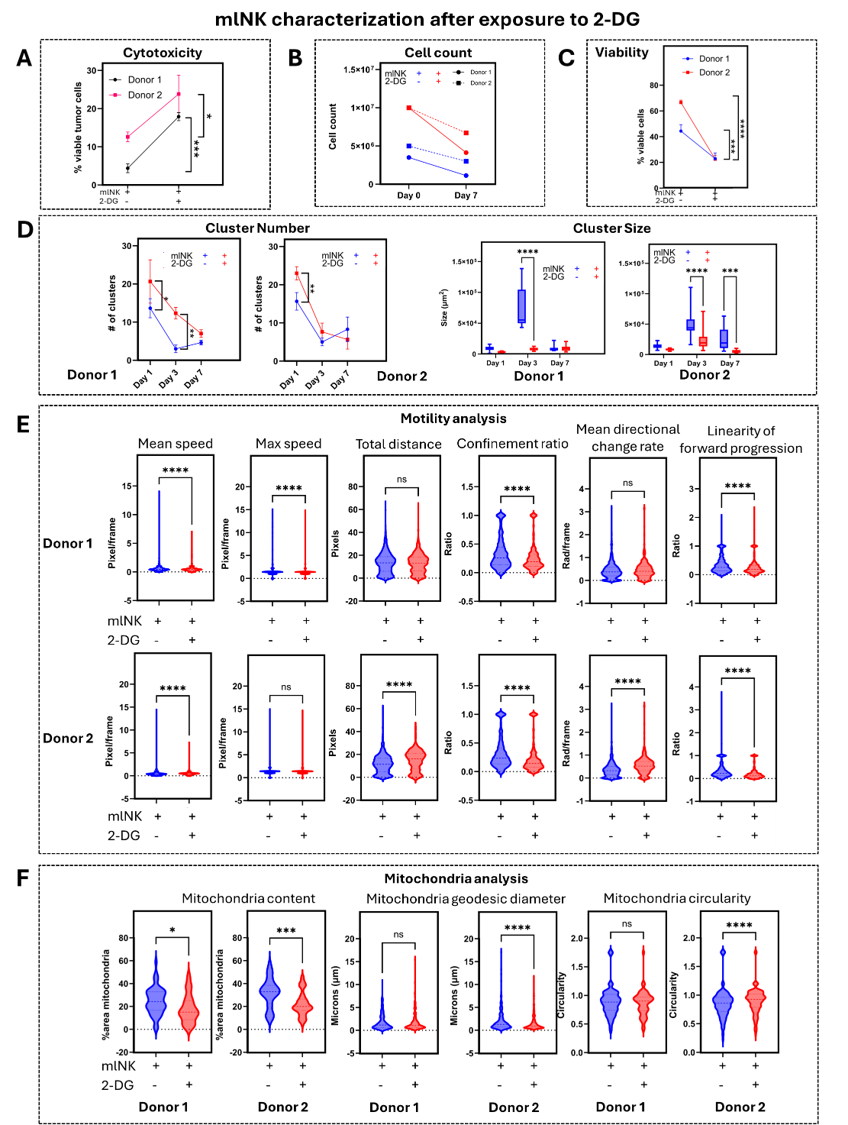

Supplement: Supplementary file 1 [file cancers-17-02288-s001.zip › Supplementary Figure S10.tif]

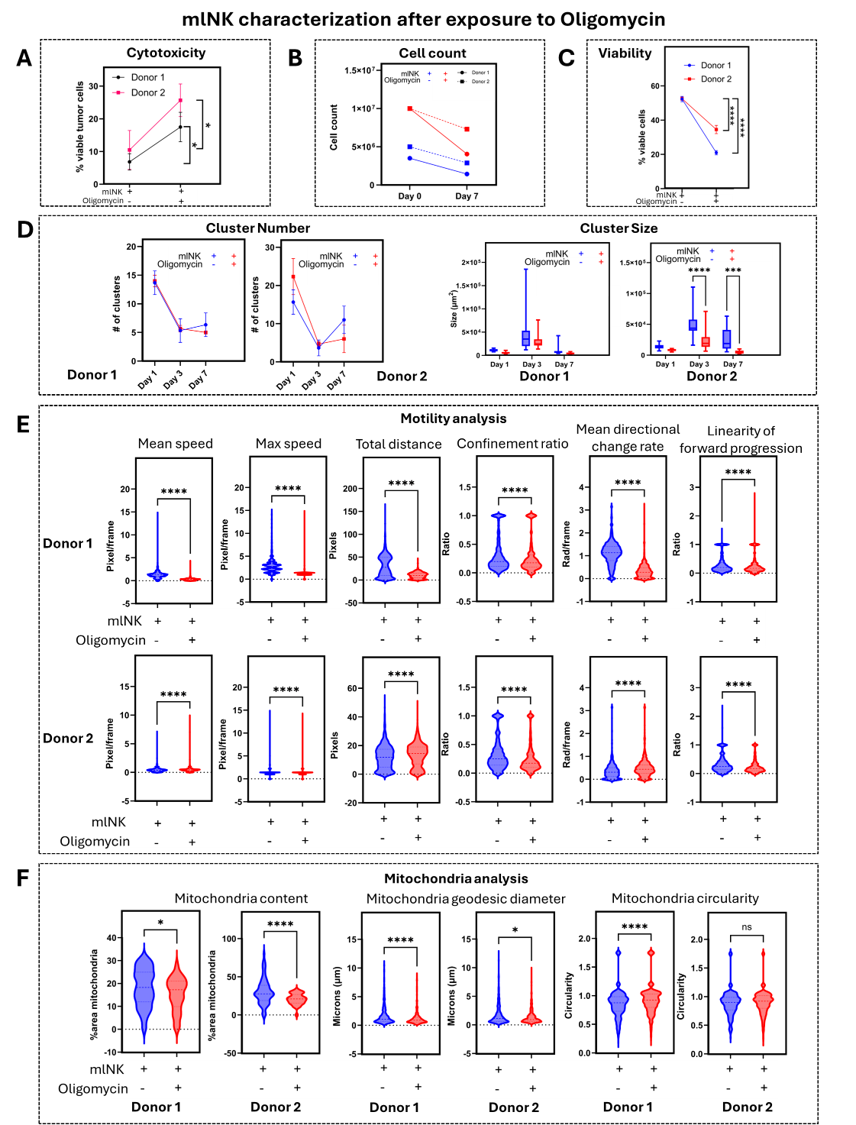

Supplement: Supplementary file 1 [file cancers-17-02288-s001.zip › Supplementary Figure S11.tif]

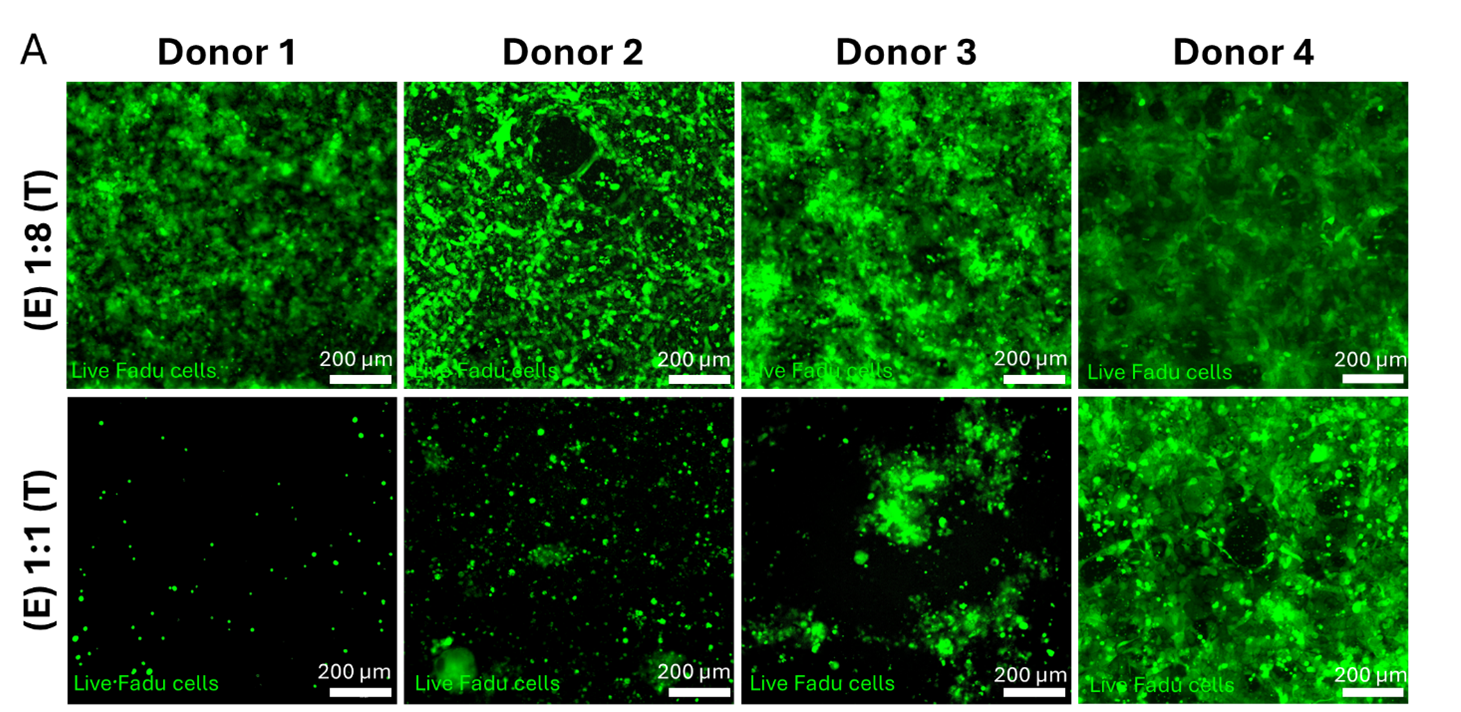

Supplement: Supplementary file 1 [file cancers-17-02288-s001.zip › Supplementary Figure S2.tif]

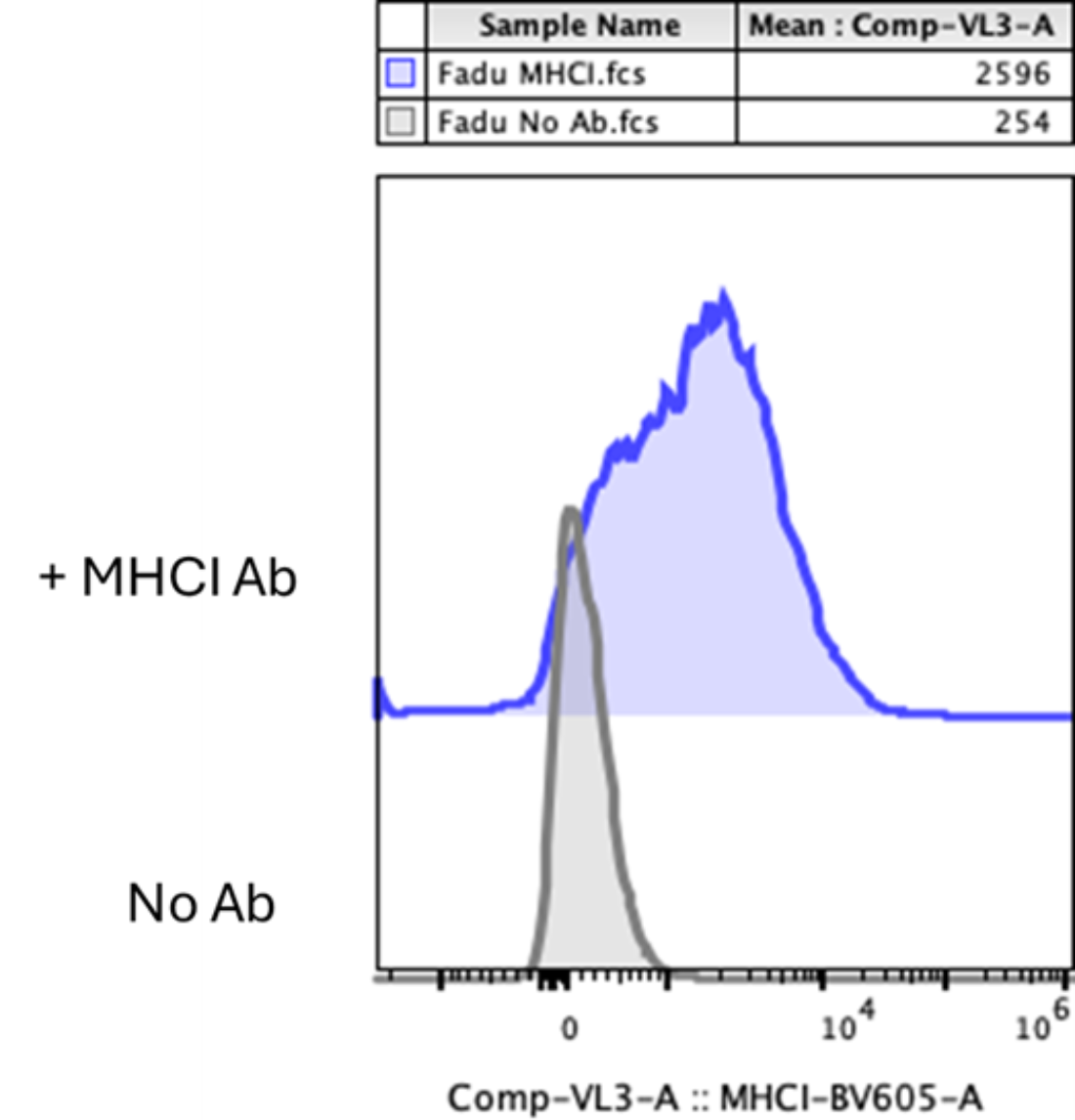

Supplement: Supplementary file 1 [file cancers-17-02288-s001.zip › Supplementary Figure S3.tif]

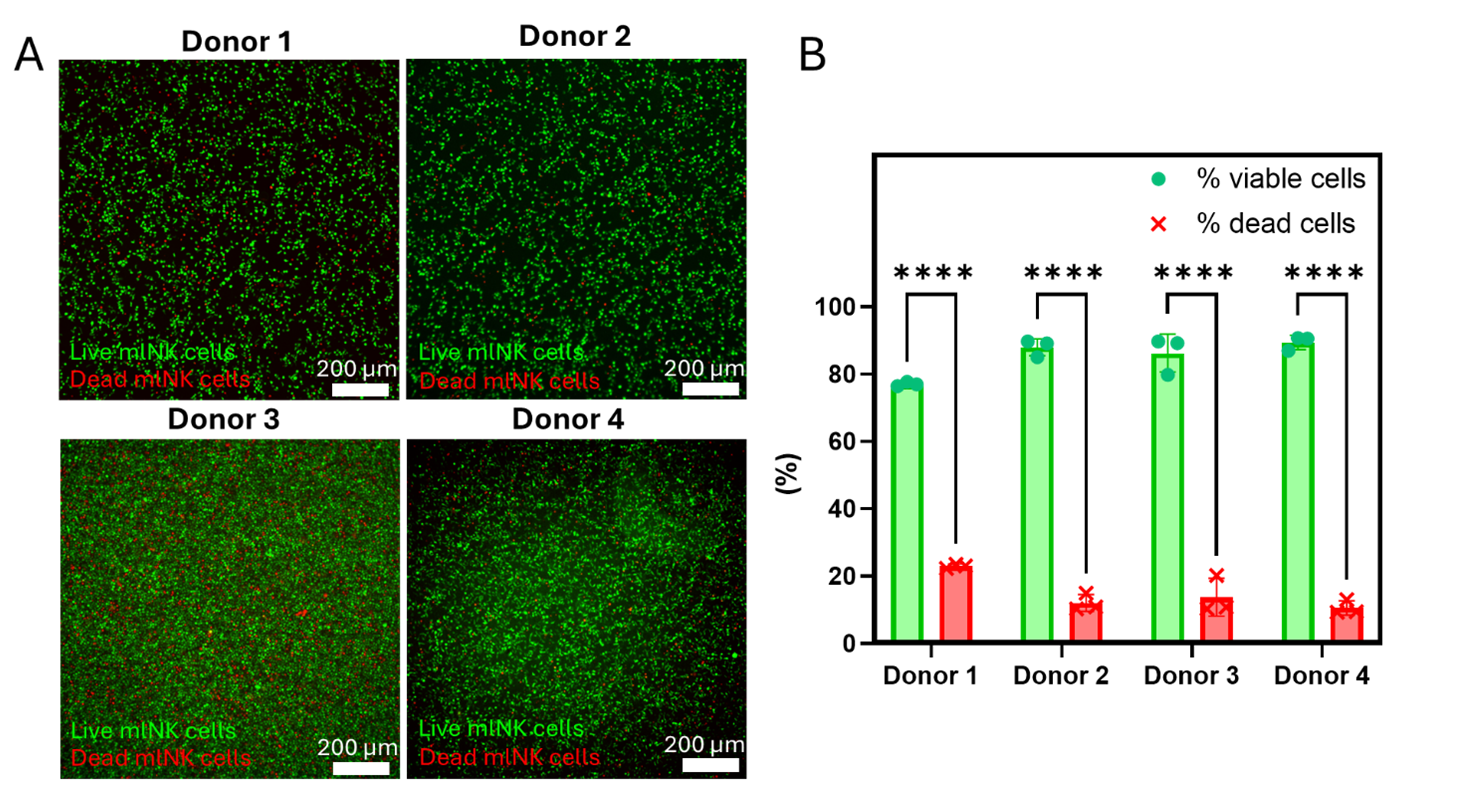

Supplement: Supplementary file 1 [file cancers-17-02288-s001.zip › Supplementary Figure S4.tif]

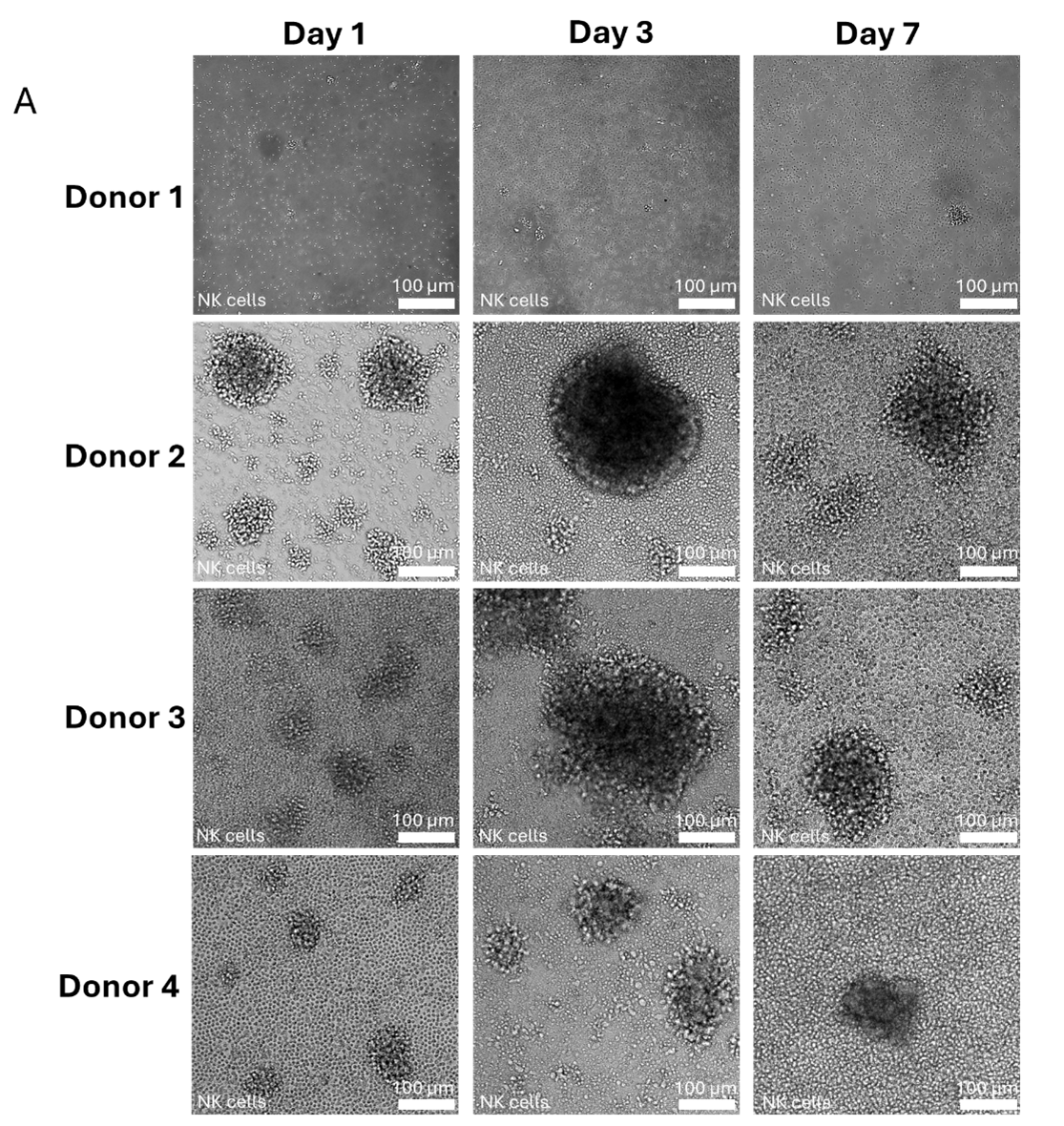

Supplement: Supplementary file 1 [file cancers-17-02288-s001.zip › Supplementary Figure S5.tif]

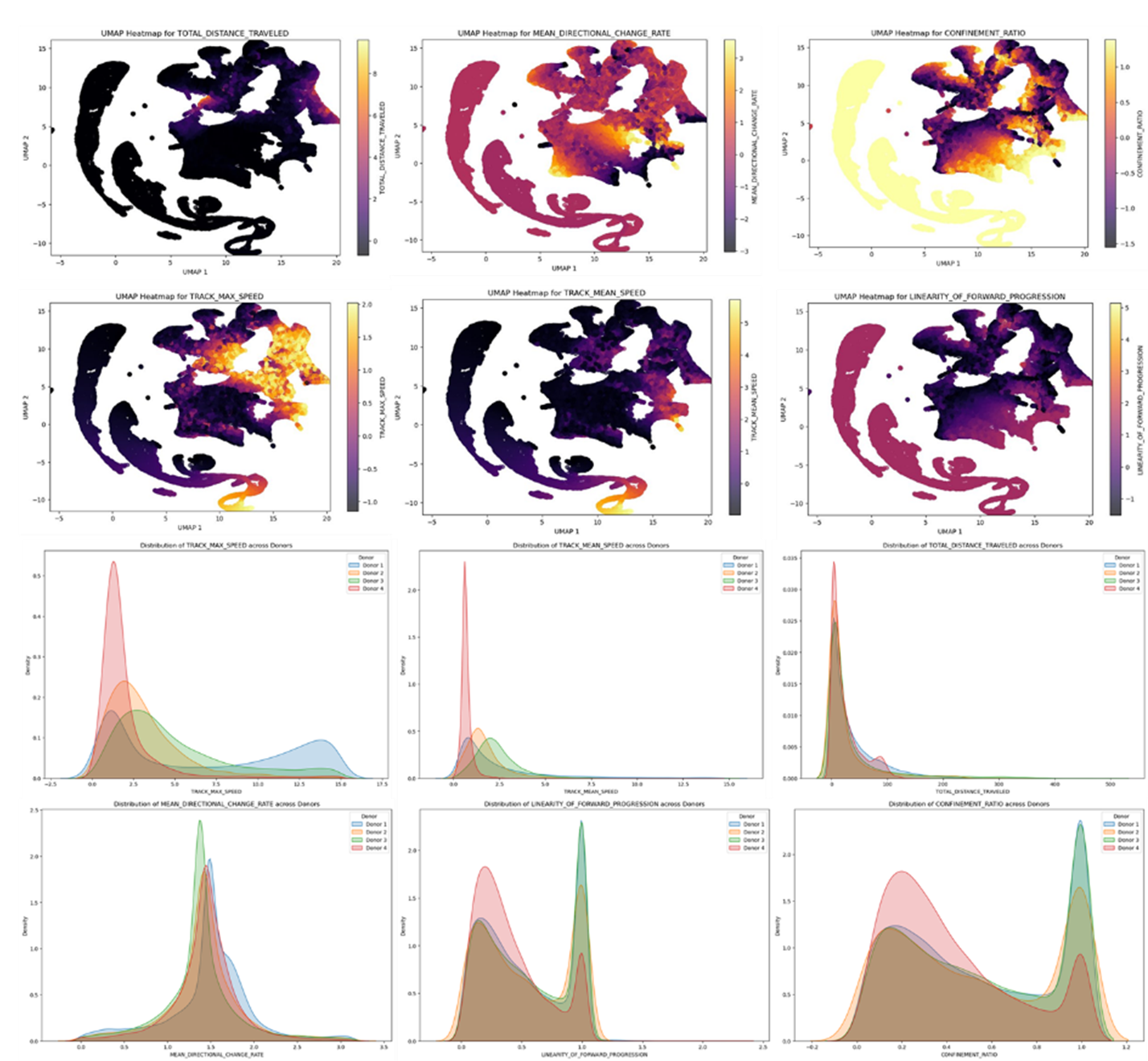

Supplement: Supplementary file 1 [file cancers-17-02288-s001.zip › Supplementary Figure S6.tif]

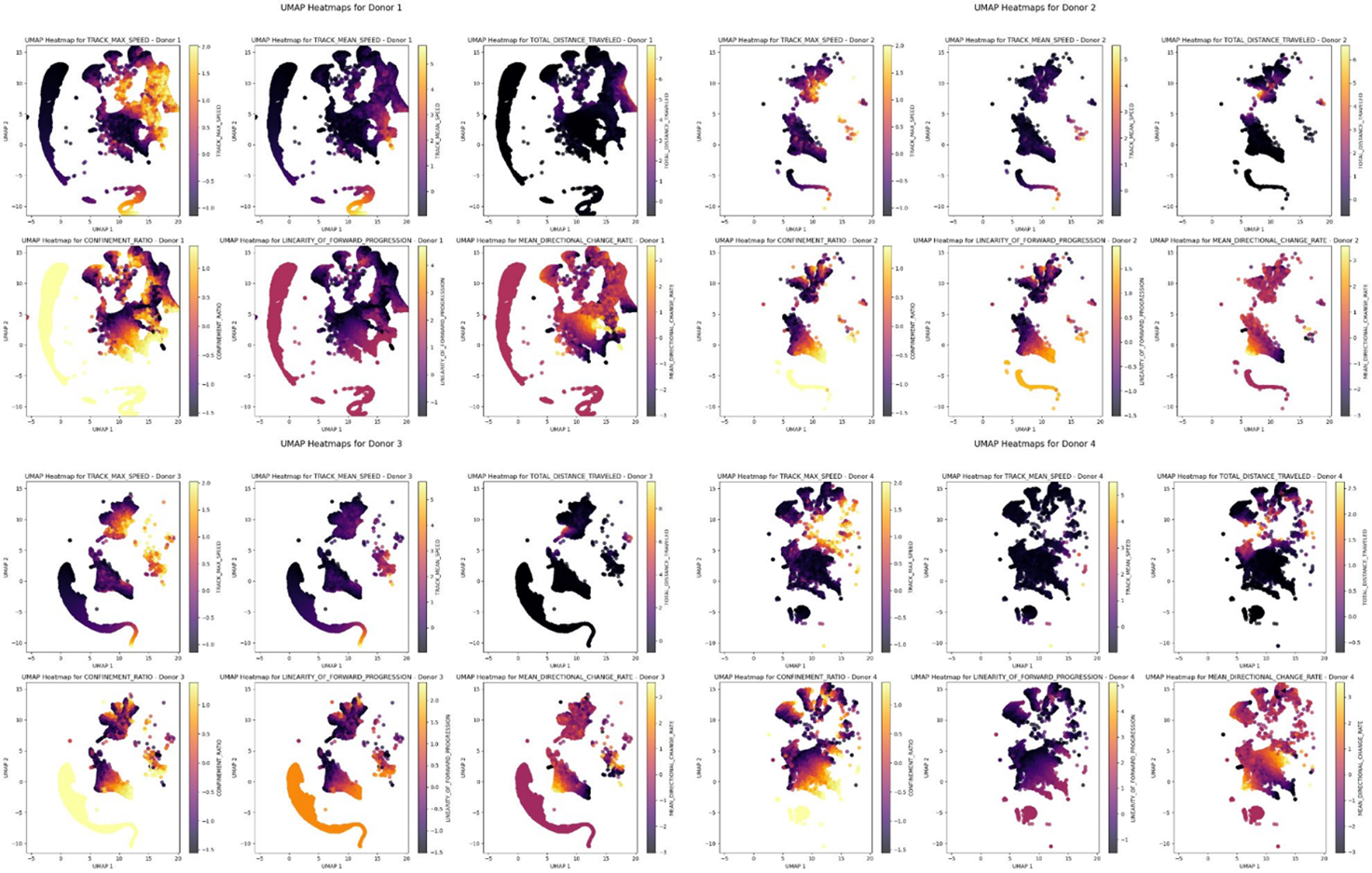

Supplement: Supplementary file 1 [file cancers-17-02288-s001.zip › Supplementary Figure S7.tif]

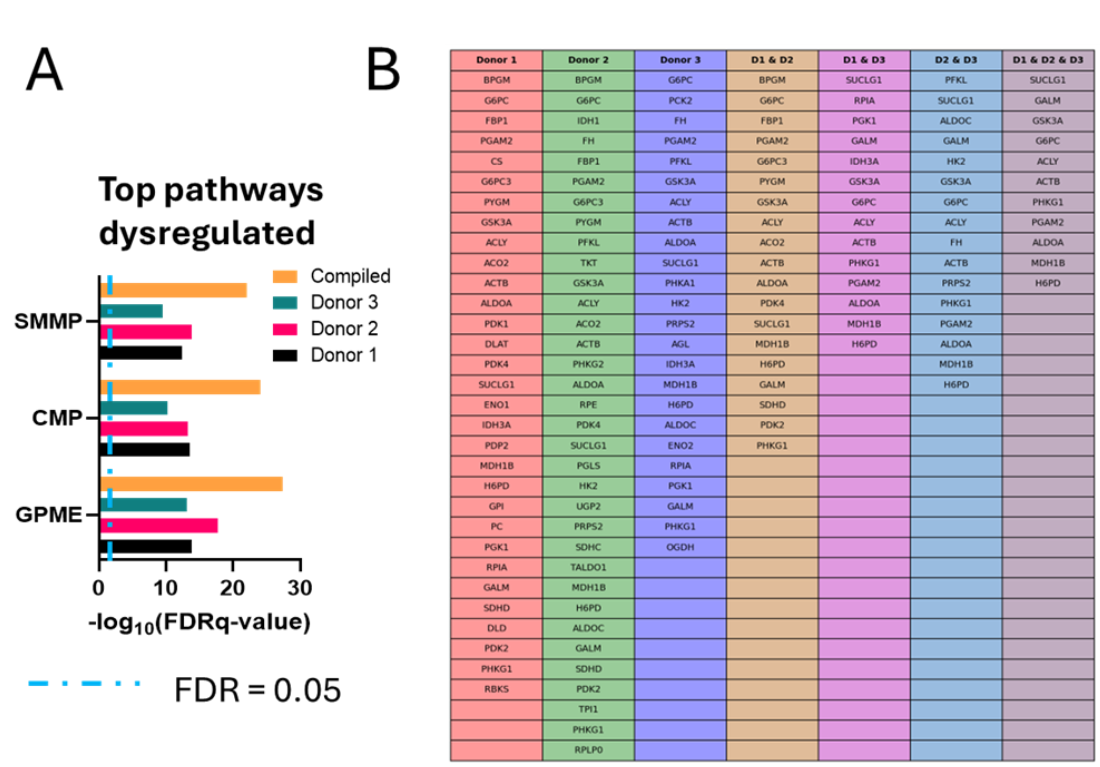

Supplement: Supplementary file 1 [file cancers-17-02288-s001.zip › Supplementary Figure S8.tif]

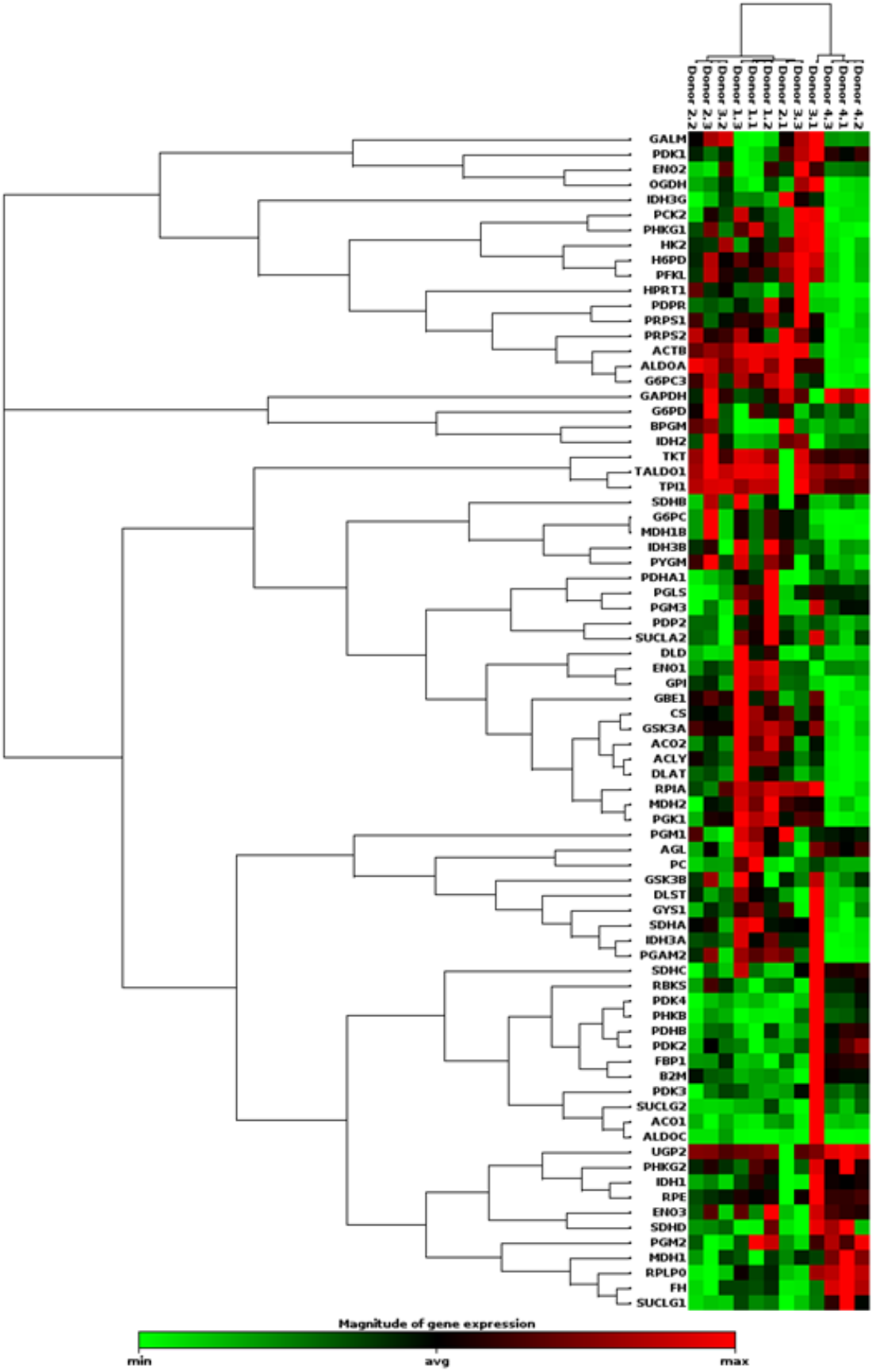

Supplement: Supplementary file 1 [file cancers-17-02288-s001.zip › Supplementary Figure S9.tif]

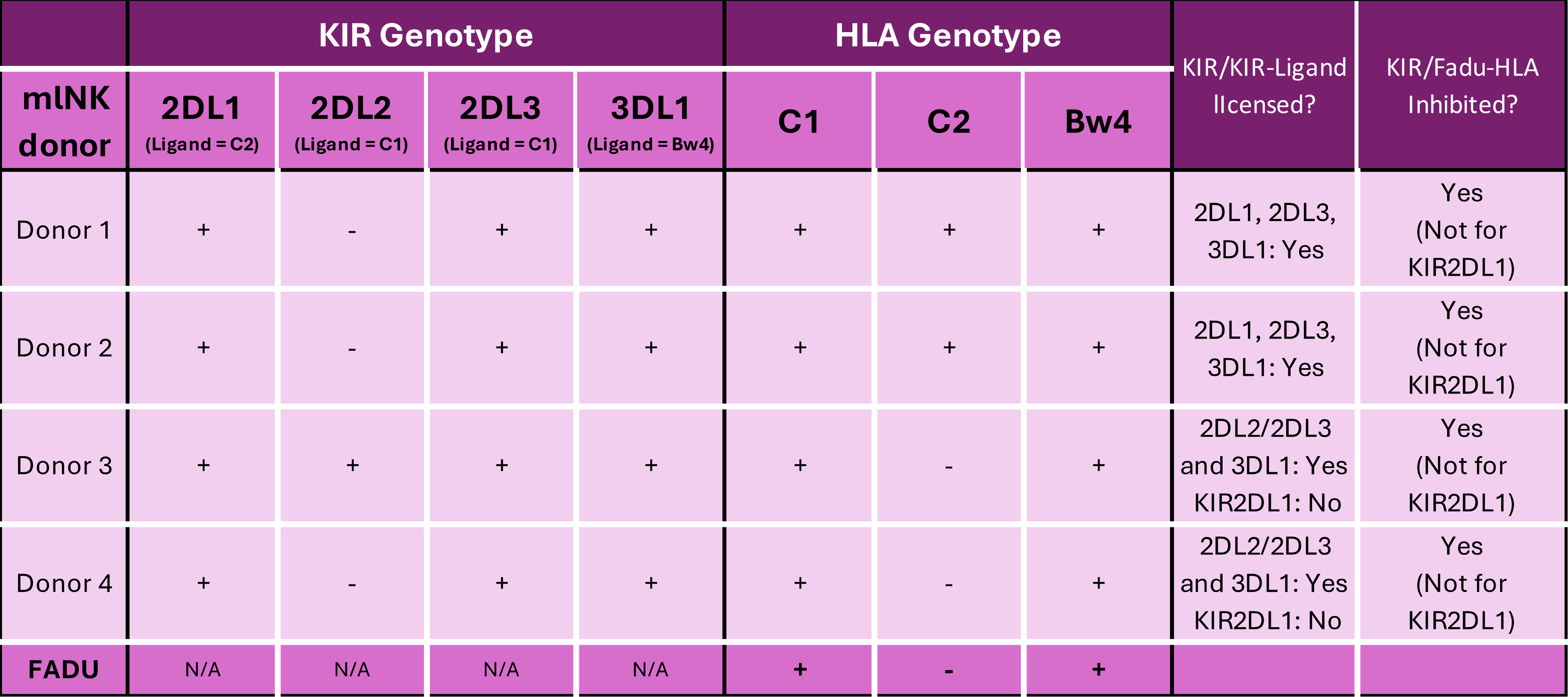

Supplement: Supplementary file 1 [file cancers-17-02288-s001.zip › Supplementary Table S1.tif]

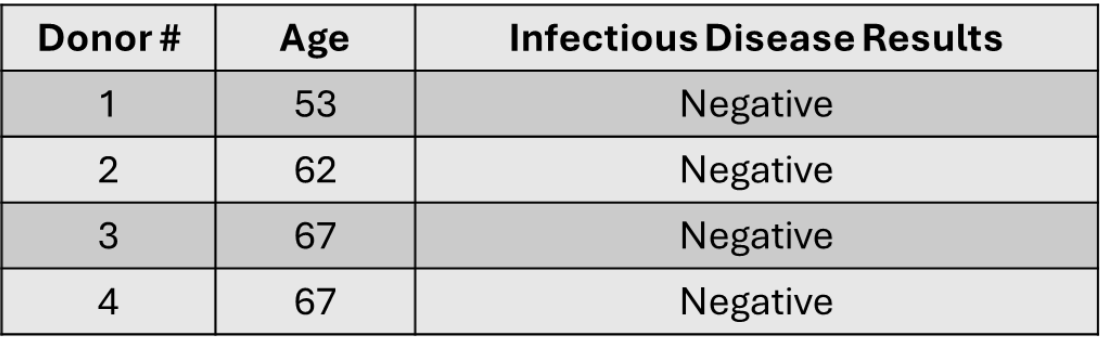

Supplement: Supplementary file 1 [file cancers-17-02288-s001.zip › Supplementary Table S2.tif]
